# Supplementary material for: Can MRI measure myelin? Systematic review, qualitative assessment, and meta-analysis of studies validating microstructural imaging with myelin histology
Source: Neuroimage. 2021 Apr 15;230:117744. doi: 10.1016/j.neuroimage.2021.117744 (PMC8063174; doi:10.1016/j.neuroimage.2021.117744)
Supplement: Supplementary Data S1 — Supplementary Raw Research Data. This is open data under the CC BY license http://creativecommons.org/licenses/by/4.0/ [file mmc1.pdf]

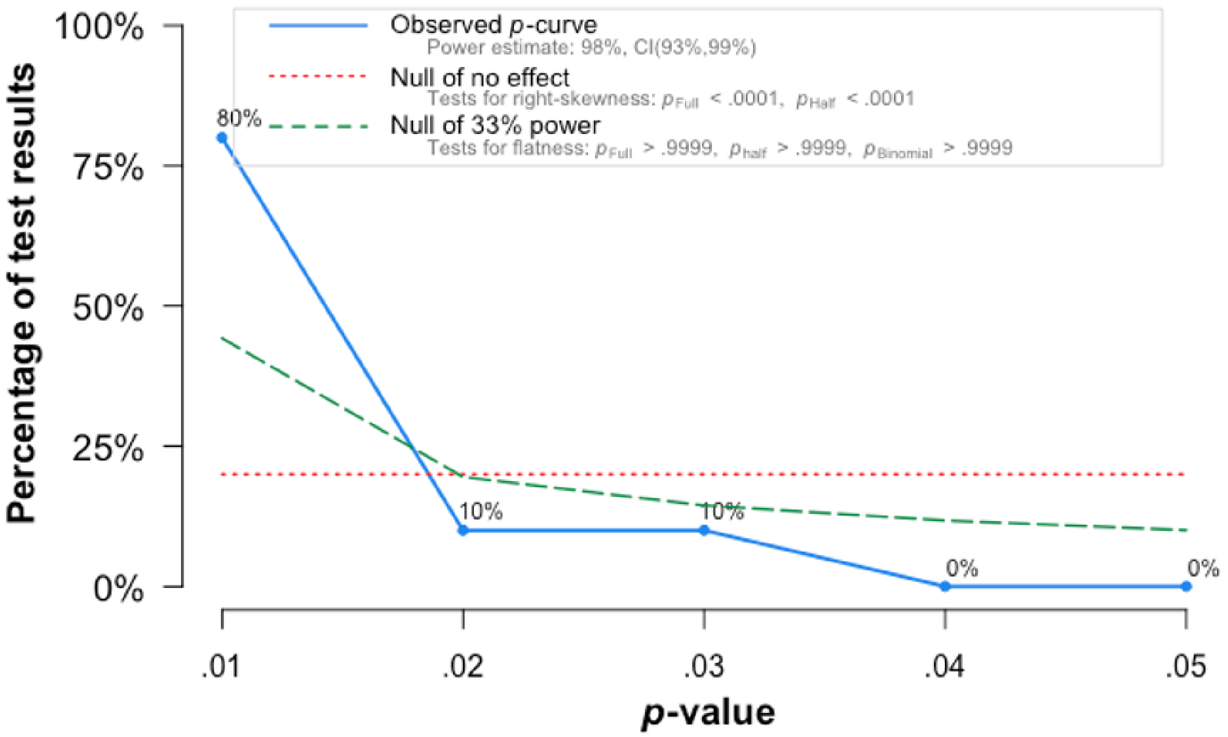

Figure 1: **p-hacking in the validation literature.** In fields where pressure to find significance biases published results p-value distributions tend to be right-skewed, i.e. to be characterized by p-values that are just below the 0.05 significance-threshold. We find evidence that the p-value distribution in the validation literature is, by contrast, left-skewed, with a majority of studies reporting p-values below 0.01.

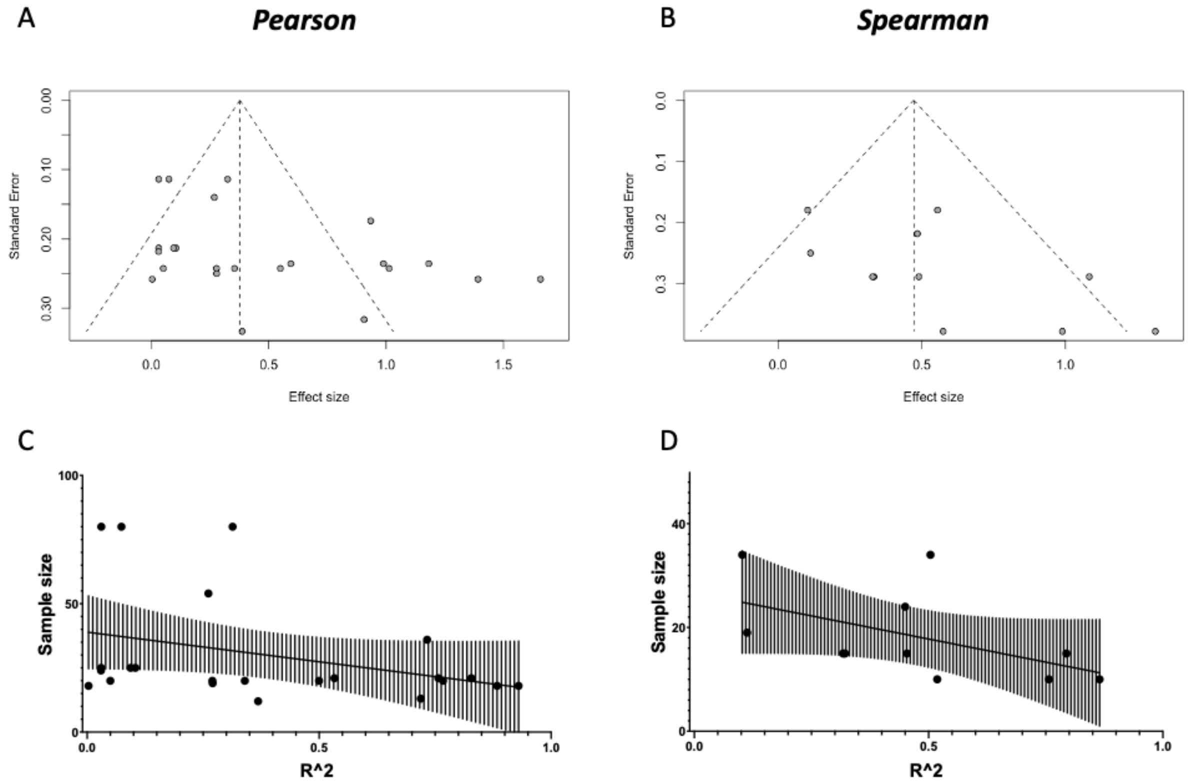

Figure 2: **Publication bias. [A and B]** Funnel plots of the studies in our quantitative meta-analyses (for Pearson and Spearman meta-analyses, respectively). We find some evidence for publication bias against low-sample size, low-effect articles (Eggers test: t = 2.419, p = 0.02471 for Pearson; t = 2.053, p = 0.07025 for Spearman). **[C and D]** To further characterize the relationship between Sample Size and Effect Size, we perform a correlation between the two, and find a relationship between higher sample sizes and lower effect sizes (Pearson: r = −0.3759, p = 0.0771; Spearman: r = −0.6451, p = 0.0365).

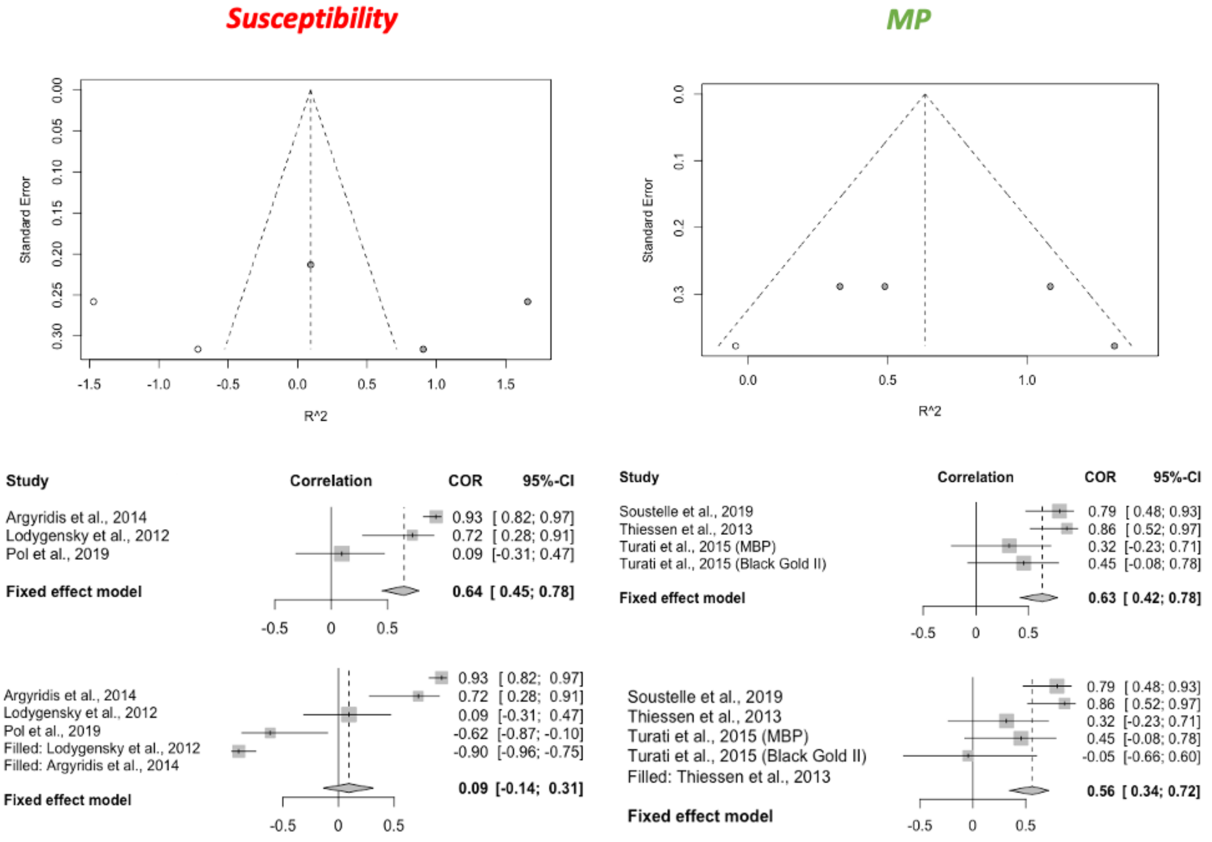

Figure 3: **Trim-and-fill correction for publication bias.** For both susceptibility and MP, we find evidence of publication bias (asymmetric funnel plots, top figures). We then use the trim-and-fill to correct for publication bias by imputing small-effect-small-sample studies that might have been performed, but not published. We then perform a meta-analysis on the imputed set of studies, and compare it to the complete case analysis in the main results.

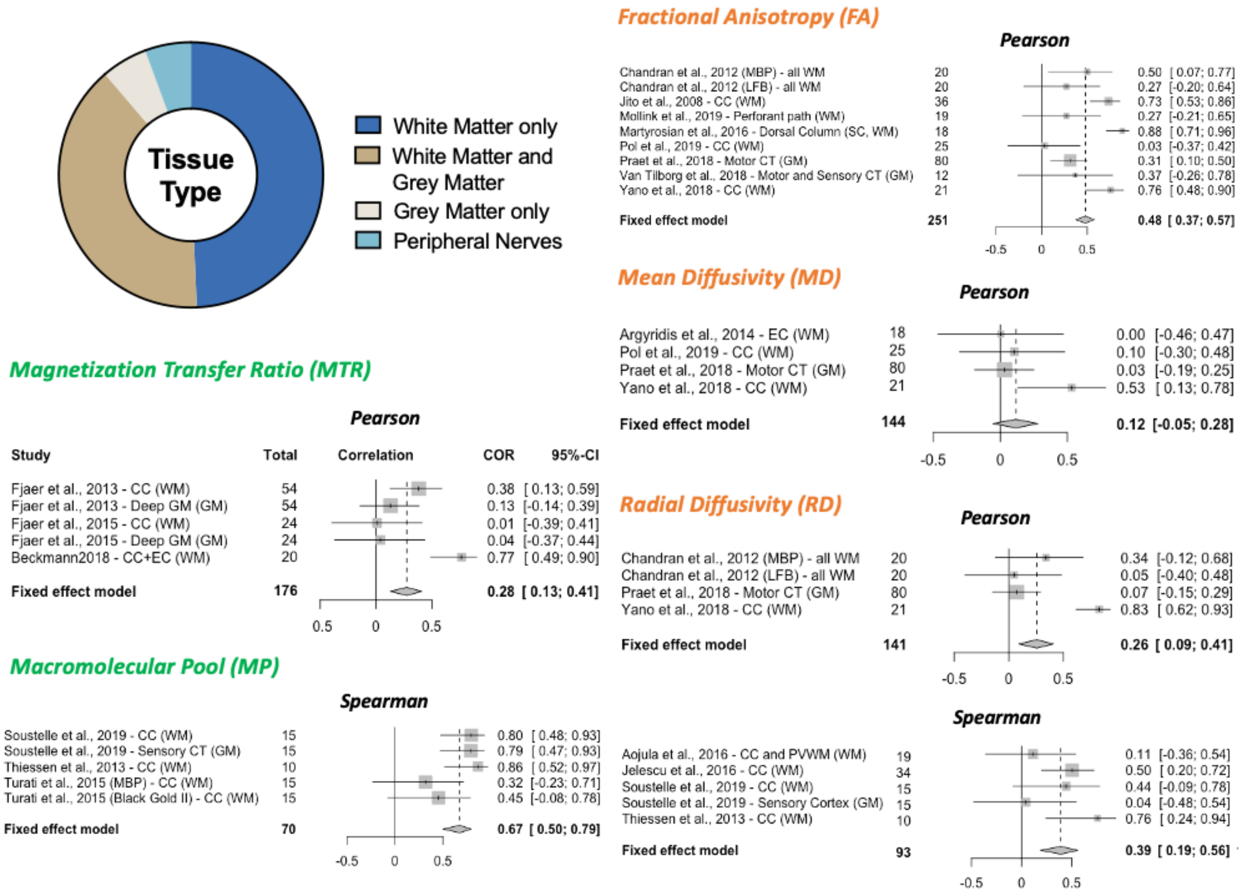

Figure 4: **Influence of tissue type on between-subject meta-analytic results.** (Top left) Within the studies considered by our systematic review (n = 71), a majority of studies investigated either White Matter regions only, or in combination with Grey Matter, whereas only a handful of studies focus on Grey Matter on its own. It is therefore unfeasible to analyse WM and GM results separately. (Top right, bottom) Because a minority of studies used for the meta-analyses include GM results, we report the results from these meta-analyses, this time splitting any WM and GM that were pooled in previous analyses. To facilitate visual inspection of this aspect of the literature, we labelled the studies according to tissue type and brain area investigated. We further confirm that excluding GM results (forest plots not shown) does not affect meta-analysis outcomes for any marker, including FA (R^2 without GM = 0.57, 95%CI = [0.44; 0.67]), MP (R^2 without GM = 0.40, 95%CI = [0.22; 0.56]) and MD (R^2 without GM = 0.23, 95%CI = [-0.03; 0.46]). Overall, choice of tissue type being investigated in different studies is unlikely to have affected the results. **Acronyms: GM**: Grey Matter, **WM**: White Matter, **CC**: Corpus Callosum, **EC**: External Capsule, **CT**: Cortex, **SC**: Spinal Cord, **PVWM**: Paraventricular White Matter.

Table 1: **Basic information of the assessed validation studies.** Provided are the species, condition/pathology studied, the tissue structure that underwent MR scanning, post-mortem time (if applicable), regions or tissue types of interest for the analysis and how they were defined. **Acronyms: ALS**: amytrophic lateral sclerosis; **APP/PS1**: Alzheimer mouse model; **Cuprizone**: Cuprizone-fed mice (D+R: demyelination and remyelination); **EAE**: allergic encephalomyelitis; **Kaolin**: Kaolin was used to induce communicating hydrocephalus; **LPS**: lipopolysaccharide-mediated animal model of MS, **MHII**: mild Hypoxic-Ischemic injury; **MPS**: mucopolysaccharidosis; **MS**: multiple sclerosis; **MSN-ChR2**: optical upregulation of Striatal medium spiny neurons **PLP-GFP**: Proteolipid protein - green fluorescent protein labelled mice; **TBI**: traumatic brain injury; **Thy1-eYFP-H**: mice that endogenously produce fluorescence signal; **TSC**: Tuberous sclerosis complex; **Shiverer**: Shiverer mice; **SCI**: spinal cord injury; **X-ALD**: X-Linked Adrenoleukodystrophy. **Anatomical structure: AC**: anterior commissure; **BG**: basal ganglia; **CC**: corpus callosum; **CP**: cerebellar peduncle; **CT**: cerebral cortex; **CST**: cortico-spinal tract; **DAWM**: diffusily abnormal white matter; **DTT**: dorsal tegmental tract; **DWMI**: diffuse white matter injury; **dGM**: deep gray matter; **GM**: gray matter; **NAWM**: normal appearing white matter; **OT**: optic tract; **PVWM**: Periventricular White Matter; **ST**: striatum; **Th**: Thalamus; **WM**: white matter; **WM-Ls**: white matter lesions.

Reference	Species	Pathology	Tissue structure	Post-mortem time	Regions / tissue types of interest	ROI definition
Choi et al. (2015)	Dog	MPS type 1 and Control	Whole brain	NA	ROIs across the brain, including WM and GM	manual
Wei et al. (2013)	Dog	Controls	Whole brain	NA	ROIs across the brain, including WM and GM	manual
Gareau et al. (2000)	Guinea pig	EAE and Controls	Brain slice	NA	NAWM in CC	manual
Bagnato et al. (2018)	Human	MS	1 cm coronal slices	4-83 hours	NAWM, DWMI, WM-Ls, thalamus, dGM, normal cortex	manual
Bot et al. (2004)	Human	MS and Controls	Cervical spinal cord	8.2±1.7 hours	WM-Ls, NAWM, WM	manual
Grussu et al. (2017)	Human	MS and Controls	Upper thoraric and upper lumbar spinal cord	not reported	GM, WM, lesional vs nonlesional	manual
Hametner et al. (2018)	Human	Controls	Whole brain	range: 72-192 hours	WM, CT, Th, BG	manual
Laule et al. (2008)	Human	MS	1 cm brain sections	not reported	WM, GM and lesions	manual
Laule et al. (2006)	Human	MS	1 cm brain sections	not reported	WM, GM and lesions	manual
Laule et al. (2011)	Human	MS	1 cm brain sections	not reported	DAWM and NAWM	manual
Moll et al. (2011)	Human	MS	Whole brain	5.8±1 hours	WM, considering distance from lesion	manual
Mollink et al. (2019)	Human	ALS and controls	Brain tissue blocks including hippocampus	range: 0.5-5 days	Perforant path	tractography / manual
Mottershead et al. (2003)	Human	MS and Controls	Spinal cord, 2 cm pieces	72±39.2	MS lesions, diffuse damage, NAMW	manual
Peters et al. (2019)	Human	Tuberculosis	Whole brain	NA	Surgically removed tissue blocks	NA
Reeves et al. (2015)	Human	Epilepsy and Control	5 mm thick piece of cortex and underlying WM	0 (samples obtained during surgery)	Normal and pathological GM and WM	manual
Righart et al. (2017)	Human	MS	Whole brain	≤ 7 hours	Left inferior frontal gyrus, superior frontal gyrus, anterior cingulate gyrus, inferior parietal gyrus, and superior temporal gyrus	atlas (Freesurfer aparc)
Schmierer et al. (2010)	Human	MS	1 cm coronal brain slices	42±32 hours	Cortical GM lesions and nonlesional CT	manual
Schmierer et al. (2004)	Human	MS	1 cm coronal brain slices	14.2±8.5 hours	WM-Ls and NAWM	manual
Schmierer et al. (2007a)	Human	MS	Coronal brain slices	15±8 (range: 4.5-43) hours, scanning: 43±22 (range 10-107) hours	WM-Ls and NAWM	manual

Continued on next page

Table 1: **Basic information of the assessed validation studies.** Provided are the species, condition/pathology studied, the tissue structure that underwent MR scanning, post-mortem time (if applicable), regions or tissue types of interest for the analysis and how they were defined. **Acronyms: ALS**: amytrophic lateral sclerosis; **APP/PS1**: Alzheimer mouse model; **Cuprizone**: Cuprizone-fed mice (D+R: demyelination and remyelination); **EAE**: allergic encephalomyelitis; **Kaolin**: Kaolin was used to induce communicating hydrocephalus; **LPS**: lipopolysaccharide-mediated animal model of MS, **MHII**: mild Hypoxic-Ischemic injury; **MPS**: mucopolysaccharidosis; **MS**: multiple sclerosis; **MSN-ChR2**: optical upregulation of Striatal medium spiny neurons **PLP-GFP**: Proteolipid protein - green fluorescent protein labelled mice; **TBI**: traumatic brain injury; **Thy1-eYFP-H**: mice that endogenously produce fluorescence signal; **TSC**: Tuberous sclerosis complex; **Shiverer**: Shiverer mice; **SCI**: spinal cord injury; **X-ALD**: X-Linked Adrenoleukodystrophy. **Anatomical structure: AC**: anterior commissure; **BG**: basal ganglia; **CC**: corpus callosum; **CP**: cerebellar peduncle; **CT**: cerebral cortex; **CST**: cortico-spinal tract; **DAWM**: diffusily abnormal white matter; **DTT**: dorsal tegmental tract; **DWMI**: diffuse white matter injury; **dGM**: deep gray matter; **GM**: gray matter; **NAWM**: normal appearing white matter; **OT**: optic tract; **PVWM**: Periventricular White Matter; **ST**: striatum; **Th**: Thalamus; **WM**: white matter; **WM-Ls**: white matter lesions.

Reference	Species	Pathology	Tissue structure	Post-mortem time	Regions / tissue types of interest	ROI definition
Schmierer et al. (2007b)	Human	MS	1 cm coronal brain slices	16±6 (range: 7-28) hours, scanning: 46±25 (range 10-107) hours	WM-Ls and NAWM	manual
Schmierer et al. (2008)	Human	MS	1 cm coronal brain slices	17±6 (range: 7-28) hours, unfixed samples: 51±28 (range: 7-108) hours	WM-Ls and NAWM	manual
Seehaus et al. (2015)	Human	Control	Whole brain	6 hours	CT and WM	NA
Seewann et al. (2009)	Human	MS	1 cm coronal brain slices	mean: 8.5 hours	WM-Ls, DAWM and NAWM	manual
Stüber et al. (2014)	Human	Controls	Brain tissue blocks (pre/postcentral gyrus, posterior occipital lobe and subthalamic nucleus)	36 and 28 hours	GM and WM	NA
van der Voorn et al. (2011)	Human	X-ALD and Controls	Coronal brain tissue sections	not reported	Areas with complete or active demyelination and NAWM	manual
Wang et al. (2015)	Human	MS	Section of cervical spinal cord	<10 hours	Voxels with positive histological staining	random selection of voxels
Warntjes et al. (2017)	Human	Controls	2 cm brain slices (one through caudate nucleus and one through thalamus)	20 hours to 3 days range (during which body was refrigerated)	GM and WM	NA
Abe et al. (2019)	Mouse	MSN-ChR2 mice	Whole brain	NA	dGM, CT	manual
Argyridis et al. (2014)	Mouse	Different neonatal stages	Whole brain	NA	EC	manual
Beckmann et al. (2018)	Mouse	Cuprizone	Whole brain	NA	CC, EC (within same ROI)	not reported
Chandran et al. (2012)	Mouse	Cuprizone	Whole brain	NA	WM	atlas-based
Chang et al. (2017b)	Mouse	Controls	Whole brain	NA	Various WM tracts	manual
Chang et al. (2017a)	Mouse	Thy1-eYFP-H mice and Controls	Whole brain	NA	Various WM tracts	manual
Duhamel et al. (2019)	Mouse	plp-GFP mice and Controls	Whole brain	NA	ROIs across the brain, including WM and GM	manual
Fatemi et al. (2011)	Mouse	Ischemic injury and control	Whole brain	NA	CC, IC, CP	manual
Fjær et al. (2013)	Mouse	Cuprizone and Controls	Whole brain	NA	CC, dGM, olfactory bulb, cerebellum and CT	manual

Continued on next page

Table 1: **Basic information of the assessed validation studies.** Provided are the species, condition/pathology studied, the tissue structure that underwent MR scanning, post-mortem time (if applicable), regions or tissue types of interest for the analysis and how they were defined. **Acronyms: ALS**: amytrophic lateral sclerosis; **APP/PS1**: Alzheimer mouse model; **Cuprizone**: Cuprizone-fed mice (D+R: demyelination and remyelination); **EAE**: allergic encephalomyelitis; **Kaolin**: Kaolin was used to induce communicating hydrocephalus; **LPS**: lipopolysaccharide-mediated animal model of MS, **MHII**: mild Hypoxic-Ischemic injury; **MPS**: mucopolysaccharidosis; **MS**: multiple sclerosis; **MSN-ChR2**: optical upregulation of Striatal medium spiny neurons **PLP-GFP**: Proteolipid protein - green fluorescent protein labelled mice; **TBI**: traumatic brain injury; **Thy1-eYFP-H**: mice that endogenously produce fluorescence signal; **TSC**: Tuberous sclerosis complex; **Shiverer**: Shiverer mice; **SCI**: spinal cord injury; **X-ALD**: X-Linked Adrenoleukodystrophy. **Anatomical structure: AC**: anterior commissure; **BG**: basal ganglia; **CC**: corpus callosum; **CP**: cerebellar peduncle; **CT**: cerebral cortex; **CST**: cortico-spinal tract; **DAWM**: diffusily abnormal white matter; **DTT**: dorsal tegmental tract; **DWMI**: diffuse white matter injury; **dGM**: deep gray matter; **GM**: gray matter; **NAWM**: normal appearing white matter; **OT**: optic tract; **PVWM**: Periventricular White Matter; **ST**: striatum; **Th**: Thalamus; **WM**: white matter; **WM-Ls**: white matter lesions.

Reference	Species	Pathology	Tissue structure	Post-mortem time	Regions / tissue types of interest	ROI definition
Fjær et al. (2015)	Mouse	EAE	Whole brain	NA	CC, dGM, olfactory bulb, cerebellum, CT	semi-automated
Jelescu et al. (2016)	Mouse	Cuprizone and controls	Whole brain	NA	CC splenium	semi-automated
Kelm et al. (2016)	Mouse	TSC / Rictor	Whole brain	NA	CC midbody, CC genu, CC splenium (SCC), AC, EC, IC	manual
Khodanovich et al. (2017)	Mouse	Cuprizone (D+R) and Controls	Whole brain	NA	GM and WM	manual
Khodanovich et al. (2019)	Mouse	Cuprizone (D+R) and Controls	Whole brain	NA	ROIs across the brain, including WM and GM	manual
Pol et al. (2019)	Mouse	Teliflunomide and Controls	Whole brain	NA	CC (caudal medial, rostral lateral, rostral medial)	manual
Praet et al. (2018)	Mouse	APP/PS1 and controls	Whole brain	NA	Motor cortex	manual on study based atlas
Soni et al. (2020)	Mouse	TBI and Controls	Whole brain	NA	Middle CC, CC-EC ipsilateral and contralateral to injury	AMBMC mouse atlas / Allen mouse atlas
Soustelle et al. (2019)	Mouse	Cuprizone and Controls	Whole brain	NA	Medial and lateral CC, CT	manual
Sundberg et al. (2010)	Mouse	SCI and Controls	Spinal cord	NA	Area centred on injury	manual
Thiessen et al. (2013)	Mouse	Cuprizone and Controls	Whole brain	NA	CC	manual
Turati et al. (2015)	Mouse	Cuprizone (D+R) and Controls	Whole brain	NA	CC	manual
West et al. (2018)	Mouse	Genetic model for hyper-/hypomyelination	Whole brain	NA	CC, AC, CT	manual
Yano et al. (2018)	Mouse	Cuprizone (D+R) and Controls	Whole brain	NA	CC	manual
Aojula et al. (2016)	Rat	Kaolin-induced hydocephalus and Controls	Whole brain	NA	CC and PVWM	manual for histology, coordinate-based for MRI
Chen et al. (2017)	Rat	SCI	3 mm spinal cord sections	NA	Fasciculus gracilis	manual
Janve et al. (2013)	Rat	LPS and control	Whole brain	NA	CC	not reported
Jespersen et al. (2010)	Rat	Controls	Whole brain	NA	ROIs across the brain, including WM and GM	manual
Kozlowski et al. (2008)	Rat	SCI and Controls	Spinal cord	NA	Fasciculus gracilis, fasciculus cuneatus, and CST	manual
Lehto et al. (2017)	Rat	Controls	Whole brain	NA	Contralateral and ipsilateral CC and DTT	manual
Lodygensky et al. (2012)	Rat	Controls	Whole brain	NA	AC, CC	manual
Martirosyan et al. (2016)	Rat	SCI	Spinal cord (T9)	NA	Epicenter of injury	manual
Oakden et al. (2015)	Rat	SCI	Caudal end of the C2 vertebra	NA	WM	manual

Continued on next page

Table 1: **Basic information of the assessed validation studies.** Provided are the species, condition/pathology studied, the tissue structure that underwent MR scanning, post-mortem time (if applicable), regions or tissue types of interest for the analysis and how they were defined. **Acronyms: ALS**: amytrophic lateral sclerosis; **APP/PS1**: Alzheimer mouse model; **Cuprizone**: Cuprizone-fed mice (D+R: demyelination and remyelination); **EAE**: allergic encephalomyelitis; **Kaolin**: Kaolin was used to induce communicating hydrocephalus; **LPS**: lipopolysaccharide-mediated animal model of MS, **MHII**: mild Hypoxic-Ischemic injury; **MPS**: mucopolysaccharidosis; **MS**: multiple sclerosis; **MSN-ChR2**: optical upregulation of Striatal medium spiny neurons **PLP-GFP**: Proteolipid protein - green fluorescent protein labelled mice; **TBI**: traumatic brain injury; **Thy1-eYFP-H**: mice that endogenously produce fluorescence signal; **TSC**: Tuberous sclerosis complex; **Shiverer**: Shiverer mice; **SCI**: spinal cord injury; **X-ALD**: X-Linked Adrenoleukodystrophy. **Anatomical structure: AC**: anterior commissure; **BG**: basal ganglia; **CC**: corpus callosum; **CP**: cerebellar peduncle; **CT**: cerebral cortex; **CST**: cortico-spinal tract; **DAWM**: diffusily abnormal white matter; **DTT**: dorsal tegmental tract; **DWMI**: diffuse white matter injury; **dGM**: deep gray matter; **GM**: gray matter; **NAWM**: normal appearing white matter; **OT**: optic tract; **PVWM**: Periventricular White Matter; **ST**: striatum; **Th**: Thalamus; **WM**: white matter; **WM-Ls**: white matter lesions.

Reference	Species	Pathology	Tissue structure	Post-mortem time	Regions / tissue types of interest	ROI definition
Odrobina et al. (2005)	Rat	Tellerium diet and Controls	Sciatic nerves	NA	Distal and proximal portion of nerve	not reported
Pun et al. (2005)	Rat	Tellerium and Controls	2 cm pieces of sciatic nerves	NA	Nerves	unclear
Schwartz et al. (2005)	Rat	Controls	Whole brain	NA	Various WM tracts	manual
Takagi et al. (2009)	Rat	Injury	Sciatic nerve	NA	Individual nerves	random selection of nerves
Tu et al. (2016)	Rat	TBI	Whole brain	NA	AC, CC, CT, CP, EC, PT, ST	manual
Underhill et al. (2011)	Rat	C6 glioma model and Controls	Whole brain	NA	ROIs across the brain, including WM and GM	manual
van Tilborg et al. (2018)	Rat	Fetal inflammation and postnatal hypoxia	Whole brain	NA	Motor cortex (M1, M2), sensory cortex	atlas-based
Wang et al. (2009)	Rat	MHII	Whole brain	NA	EC (ipsilesional and contralesional)	manual
Webb et al. (2003)	Rat	Nerve injury and Controls	Sciatic nerve	NA	Sections of individual nerves	manual
Hakkarainen et al. (2016)	Rats	Controls	Whole brain	NA	ROIs across the brain	not reported
Jito et al. (2008)	Rats	6 postnatal stages	Whole brain	NA	CC	manual
Kozlowski et al. (2014)	Rats	SCI	Spinal cord	NA	Dorsal column	manual

Table 2: **Information on MRI imaging of the assessed validation studies.** Provided are imaging modality, field strength in Tesla (T), imaging resolution in terms of voxel size, and the tissue state and temperature during scanning. **Acronyms: Imaging: DWI: Diffusion-weighted imaging**: **AK**: axial kurtosis; **AI**: Anisotropy Index (tADC/lADC); **DBSI-RD**: diffusion basis spectrum imaging - based radial diffusivity; **DK**: diffusion kurtosis metrics; **DT**: diffusion tensor metrics; **FA**: fractional anisotropy (from diffusion tensor model); **lADC**: longitudinal apparent diffusion coefficient (not modelled with tensor); **MD**: mean diffusivity (from diffusion tensor model); **MK**: mean kurtosis; **RD**: radial / transverse diffusivity (from diffusion tensor model); **RK**: radial kurtosis; **SDI**: diffusion standard deviation index; **tADC**: transverse apparent diffusion coefficient (not modelled with tensor); **Relaxometry**: **MWF**: myelin water fraction; **R1**: longitudinal relaxation rate; **R2***: effective transverse relaxation rate; **RAFF4**: Relaxation Along a Fictitious Field in the rotating frame of rank 4; **T1**: longitudinal relaxation time; **T2**: transverse relaxation time; **T2***: effective transverse relaxation time; **MT: magnetisation transfer**: **BPF**: bound pool fraction; **F**: pool size ratio; **Fb**: macromolecular proton fraction; **ih-MTR**: MTR from inhomogeneous MT; **M0b**: fraction of magnetization that resides in the semi-solid pool and undergoes MT exchange; **MP**: macromolecular pool; **MPF**: macromolecular proton fraction; **MTR**: magnetisation transfer ratio; **PSR**: Macromolecular-to-free-water pool-size-ratio; **STE-MT**: MTR based on short echo time imaging; T1sat: T1 of saturated pool; **UTE-MTR**: MTR based on ultrashort echo time imaging; **QSM**: quantitative susceptibility mapping; **Others: rSPF**: relative semi-solid proton fraction from an 3D ultrashort echo time (UTE) sequence within an appropriate water suppression condition; **T1w/T2w**: ratio of image intensity in a T1-weighted vs T2-weighted acquisition.

Reference	Modality	Metrics	T	Resolution (mm)	Tissue state	Temperature
Abe et al. (2019)	DWI	AD, FA, RD	7	0.125 x 0.125 x 0.125 mm	perfusion fixed	not reported
Aojula et al. (2016)	DWI	AD, FA, MD, RD	7	0.195 x 0.195 x 1mm	in vivo	body temperature
Argyridis et al. (2014)	DWI	MD	9.4	unclear	perfusion fixed	not reported
Chandran et al. (2012)	DWI	FA, RD	7	0.2 x 0.2 x 1 mm	in vivo	body temperature
Chang et al. (2017b)	DWI	FA, AD, RD	11.7	0.1 mm isotropic	perfusion fixed	28°C
Chang et al. (2017a)	DWI	FA, AD, RD, MD	11.7	0.1 mm isotropic	perfusion fixed	28°C
Choi et al. (2015)	DWI	FA, RD	7	0.1 mm isotropic	fixed	not reported

Continued on next page

Table 2: **Information on MRI imaging of the assessed validation studies.** Provided are imaging modality, field strength in Tesla (T), imaging resolution in terms of voxel size, and the tissue state and temperature during scanning. **Acronyms: Imaging: DWI: Diffusion-weighted imaging**: **AK**: axial kurtosis; **AI**: Anisotropy Index (tADC/lADC); **DBSI-RD**: diffusion basis spectrum imaging - based radial diffusivity; **DK**: diffusion kurtosis metrics; **DT**: diffusion tensor metrics; **FA**: fractional anisotropy (from diffusion tensor model); **lADC**: longitudinal apparent diffusion coefficient (not modelled with tensor); **MD**: mean diffusivity (from diffusion tensor model); **MK**: mean kurtosis; **RD**: radial / transverse diffusivity (from diffusion tensor model); **RK**: radial kurtosis; **SDI**: diffusion standard deviation index; **tADC**: transverse apparent diffusion coefficient (not modelled with tensor); **Relaxometry**: **MWF**: myelin water fraction; **R1**: longitudinal relaxation rate; **R2***: effective transverse relaxation rate; **RAFF4**: Relaxation Along a Fictitious Field in the rotating frame of rank 4; **T1**: longitudinal relaxation time; **T2**: transverse relaxation time; **T2***: effective transverse relaxation time; **MT: magnetisation transfer**: **BPF**: bound pool fraction; **F**: pool size ratio; **Fb**: macromolecular proton fraction; **ih-MTR**: MTR from inhomogeneous MT; **M0b**: fraction of magnetization that resides in the semi-solid pool and undergoes MT exchange; **MP**: macromolecular pool; **MPF**: macromolecular proton fraction; **MTR**: magnetisation transfer ratio; **PSR**: Macromolecular-to-free-water pool-size-ratio; **STE-MT**: MTR based on short echo time imaging; T1sat: T1 of saturated pool; **UTE-MTR**: MTR based on ultrashort echo time imaging; **QSM**: quantitative susceptibility mapping; **Others: rSPF**: relative semi-solid proton fraction from an 3D ultrashort echo time (UTE) sequence within an appropriate water suppression condition; **T1w/T2w**: ratio of image intensity in a T1-weighted vs T2-weighted acquisition.

Reference	Modality	Metrics	T	Resolution (mm)	Tissue state	Temperature
Grussu et al. (2017)	DWI	AD, FA, MD, RD	9.4	0.2 x 0.2 x 2 mm	fixed	35°C
Janve et al. (2013)	DWI	AD, FA, RD	9.4	0.167 x 0.167 x 0.167 mm	perfusion fixed	not reported
Jelescu et al. (2016)	DWI	RD, RK	7	0.112 x 0.112 x 0.8 mm	in vivo	body temperature
Jespersen et al. (2010)	DWI	FA*	16.4	0.1 x 0.1 x 0.5 mm	perfusion fixed	21°C
Jito et al. (2008)	DWI	FA	7	0.117 x 0.24 x 1 mm	in vivo	body temperature
Kelm et al. (2016)	DWI	FA, MD, RD, MK, AK, RK	15.2	0.15 x 0.15 x 0.15 mm	perfusion fixed	17 ± 0.5 °C
Kozlowski et al. (2008)	DWI	FA, AD, MD, RD	7	1 x 1 x 1 mm	perfusion fixed	not reported
Kozlowski et al. (2014)	DWI	FA	7	1 x 1 x 1 mm (only ex vivo)	in vivo (relaxometry) and fixed (DWI)	body temperature / not reported
Lehto et al. (2017)	DWI	AD, FA, MD, RD	7	0.125 x 0.125 x 0.5 mm	in vivo	body temperature
Martirosyan et al. (2016)	DWI	FA	7	0.195 x 0.195 x 10 mm	perfusion fixed	not reported
Moll et al. (2011)	DWI	AD, FA, MD, RD	1.5	1.9 1.9 3 mm	in situ / fixed	body temperature
Mollink et al. (2019)	DWI	FA, MD, RD, AD	11.7	0.4 x 0.4 x 0.4 mm resolution.	fixed	not reported
Mottershead et al. (2003)	DWI	ADC, SDI	7	Single 1.5 mm slices, in plane resolution between 0.055 and 0.068 mm	fresh	29-38°C
Oakden et al. (2015)	DWI	AD, RD	1.5	0.2 0.2 x 1 mm	in vivo / fixed	body temperature
Peters et al. (2019)	DWI	FA, MD	3	1.72 x 1.72 x 2.2 mm	in vivo	body temperature
Pol et al. (2019)	DWI	FA, MD	9.4	0.078 0.078 0.250 mm	in vivo	body temperature
Praet et al. (2018)	DWI	AD, AK, DT, DK, FA, MD, MK, RD, RK	7	0.000214 x 0.000214 x 0.2 mm	in vivo	body temperature (37-37.3°C)
Schmierer et al. (2007b)	DWI	FA, MD	1.5	2.5 x 2.5 x 5 mm	fresh	17.5 - 25.1°C
Schmierer et al. (2008)	DWI	AD, FA, MD, RD	1.5	0.25 x 0.25 x 5 mm	fresh / fixed	22.5°C
Schwartz et al. (2005)	DWI	AI, iADC, tADC	9.4	0.039 x 0.039 x 0.5 mm	perfusion fixed	20°C
Seehaus et al. (2015)	DWI	FA	9.4	0.34 x 0.34 x 0.34 mm	fixed	30 °C
Seewann et al. (2009)	DWI	FA, ADC	1.5	2 x 2 x 8 mm	fixed	not reported
Soustelle et al. (2019)	DWI	RD	7	0.1 x 0.1 x 0.750 mm	perfusion fixed	room temperature (20°C)
Sundberg et al. (2010)	DWI	AD, FA, RD	7	0.2 x 0.2 x 1 mm	in vivo	37°C
Takagi et al. (2009)	DWI	FA	7	0.31 x 0.31 x 0.94 mm	excised	not reported
Thiessen et al. (2013)	DWI	AD, FA, RD	7	0.098 0.098 0.750 mm	perfusion fixed	room temperature
Tu et al. (2016)	DWI	AD, FA, MD, RD, MTR at different ppm	7	DWI: 0.2 x 0.2 x 0.2 mm; MT: 0.2 x 0.2 x 0.5 mm;	in vivo	body temperature

Continued on next page

Table 2: **Information on MRI imaging of the assessed validation studies.** Provided are imaging modality, field strength in Tesla (T), imaging resolution in terms of voxel size, and the tissue state and temperature during scanning. **Acronyms: Imaging: DWI: Diffusion-weighted imaging**: **AK**: axial kurtosis; **AI**: Anisotropy Index (tADC/lADC); **DBSI-RD**: diffusion basis spectrum imaging - based radial diffusivity; **DK**: diffusion kurtosis metrics; **DT**: diffusion tensor metrics; **FA**: fractional anisotropy (from diffusion tensor model); **lADC**: longitudinal apparent diffusion coefficient (not modelled with tensor); **MD**: mean diffusivity (from diffusion tensor model); **MK**: mean kurtosis; **RD**: radial / transverse diffusivity (from diffusion tensor model); **RK**: radial kurtosis; **SDI**: diffusion standard deviation index; **tADC**: transverse apparent diffusion coefficient (not modelled with tensor); **Relaxometry**: **MWF**: myelin water fraction; **R1**: longitudinal relaxation rate; **R2***: effective transverse relaxation rate; **RAFF4**: Relaxation Along a Fictitious Field in the rotating frame of rank 4; **T1**: longitudinal relaxation time; **T2**: transverse relaxation time; **T2***: effective transverse relaxation time; **MT: magnetisation transfer**: **BPF**: bound pool fraction; **F**: pool size ratio; **Fb**: macromolecular proton fraction; **ih-MTR**: MTR from inhomogeneous MT; **M0b**: fraction of magnetization that resides in the semi-solid pool and undergoes MT exchange; **MP**: macromolecular pool; **MPF**: macromolecular proton fraction; **MTR**: magnetisation transfer ratio; **PSR**: Macromolecular-to-free-water pool-size-ratio; **STE-MT**: MTR based on short echo time imaging; T1sat: T1 of saturated pool; **UTE-MTR**: MTR based on ultrashort echo time imaging; **QSM**: quantitative susceptibility mapping; **Others: rSPF**: relative semi-solid proton fraction from an 3D ultrashort echo time (UTE) sequence within an appropriate water suppression condition; **T1w/T2w**: ratio of image intensity in a T1-weighted vs T2-weighted acquisition.

Reference	Modality	Metrics	T	Resolution (mm)	Tissue state	Temperature
van der Voorn et al. (2011)	DWI	ADC, FA	1.5	2 x 2 x 8 mm	fixed	room temperature (20-22°C)
van Tilborg et al. (2018)	DWI	FA, RD	9.4	0.15 x 0.15 x 0.148 mm	perfusion fixed	not reported
Wang et al. (2009)	DWI	AD, FA, RD, "Trace"	7	Varying resolution: 0.250 x 0.250 x 0.5 mm for some scans, 0.313 x 0.313 x 0.7 mm for others	in vivo	body temperature
Wang et al. (2015)	DWI	DBSI-RD	4.7	0.250 x 0.250 x 0.5 mm	fixed	17°C
Wei et al. (2013)	DWI	FA, RD	7	0.1 mm isotropic	perfusion fixed	not reported
Yano et al. (2018)	DWI	FA, MD, RD	7	0.125 0.125 0.125 mm	perfusion fixed	not reported
Beckmann et al. (2018)	MT	MTR	7	0.094 0.094 x 0.5 mm	in vivo	body temperature
Bot et al. (2004)	MT	MTR	4.7	0.007 x 0.007 x 1 mm	fixed	not reported
Duhamel et al. (2019)	MT	ih-MTR	11.75	0.3125 x 0.3125 x 1 mm	in vivo	37°C
Fatemi et al. (2011)	MT	MTR	9.4	0.083 x 0.81 x 0.8 mm	in vivo	body temperature
Fjær et al. (2013)	MT	MTR	7	0.2 x 0.2 x 0.23 mm	in vivo	body temperature
Fjær et al. (2015)	MT	MTR	7	0.2 x 0.2 x 0.23 mm	in vivo	body temperature
Gareau et al. (2000)	MT	MTR	4	0.160 x 0.3 x 0.5 mm	in vivo	body temperature
Hakkarainen et al. (2016)	MT	MTR	9.4	0.0293 x 0.0293 x 0.7 mm	perfusion fixed	not reported
Janve et al. (2013)	MT	MP (PSR)	9.4	0.167 x 0.167 x 0.167 mm	perfusion fixed	not reported
Jelescu et al. (2016)	MT	MTR	7	0.112 x 0.112 x 0.8 mm	in vivo	body temperature
Kelm et al. (2016)	MT	MP (PSR)	15.2	0.15 x 0.15 x 0.15 mm	perfusion fixed	17 ± 0.5 °C
Khodanovich et al. (2017)	MT	MP ('MPF')	11.7	0.1 x 0.1 x 0.5 mm	fixed	body temperature
Khodanovich et al. (2019)	MT	MP ('MPF')	11.7	0.1 x 0.1 x 0.5 mm	fixed	body temperature
Lehto et al. (2017)	MT	T1sat, MTR	7	0.125 x 0.125 x 0.5 mm	in vivo	body temperature
Moll et al. (2011)	MT	MTR	1.5	0.9 0.9 3 mm	in situ / fixed	body temperature
Mottershead et al. (2003)	MT	MTR	7	Single 1.5 mm slices, in plane resolution between 0.055 and 0.068 mm	fresh	29-38°C
Odrobina et al. (2005)	MT	MTR, MP('M0b')	1.5	not reported	fixed	room temperature (20°C)
Reeves et al. (2015)	MT	MTR	9.4	0.136 x 0.136 x 0.500 mm	fixed	not reported
Schmierer et al. (2010)	MT	MTR	9.4	not reported	fixed	not reported
Schmierer et al. (2004)	MT	MTR	1.5	0.938 x 0.938 x 5 mm	fresh	mean 25.0°C (SD: 2.9°C)
Schmierer et al. (2007a)	MT	MTR, MP('fb')	1.5	0.938 x 0.938 x 5 mm	fresh	room temperature
Schmierer et al. (2008)	MT	MTR, MP('fb')	1.5	0.94 x 0.94 x 5 mm	fresh / fixed	22.5°C
Seewann et al. (2009)	MT	MTR	1.5	1 x 1 x 5 mm	fixed	not reported
Soustelle et al. (2019)	MT	MP('f')	7	0.1 x 0.1 x 0.750 mm	perfusion fixed	room temperature (20°C)
Thiessen et al. (2013)	MT	MP('f'), MTR	7	0.098 0.098 0.750 mm	perfusion fixed	body temperature

Continued on next page

Table 2: **Information on MRI imaging of the assessed validation studies.** Provided are imaging modality, field strength in Tesla (T), imaging resolution in terms of voxel size, and the tissue state and temperature during scanning. **Acronyms: Imaging: DWI: Diffusion-weighted imaging**: **AK**: axial kurtosis; **AI**: Anisotropy Index (tADC/lADC); **DBSI-RD**: diffusion basis spectrum imaging - based radial diffusivity; **DK**: diffusion kurtosis metrics; **DT**: diffusion tensor metrics; **FA**: fractional anisotropy (from diffusion tensor model); **lADC**: longitudinal apparent diffusion coefficient (not modelled with tensor); **MD**: mean diffusivity (from diffusion tensor model); **MK**: mean kurtosis; **RD**: radial / transverse diffusivity (from diffusion tensor model); **RK**: radial kurtosis; **SDI**: diffusion standard deviation index; **tADC**: transverse apparent diffusion coefficient (not modelled with tensor); **Relaxometry**: **MWF**: myelin water fraction; **R1**: longitudinal relaxation rate; **R2***: effective transverse relaxation rate; **RAFF4**: Relaxation Along a Fictitious Field in the rotating frame of rank 4; **T1**: longitudinal relaxation time; **T2**: transverse relaxation time; **T2***: effective transverse relaxation time; **MT: magnetisation transfer**: **BPF**: bound pool fraction; **F**: pool size ratio; **Fb**: macromolecular proton fraction; **ih-MTR**: MTR from inhomogeneous MT; **M0b**: fraction of magnetization that resides in the semi-solid pool and undergoes MT exchange; **MP**: macromolecular pool; **MPF**: macromolecular proton fraction; **MTR**: magnetisation transfer ratio; **PSR**: Macromolecular-to-free-water pool-size-ratio; **STE-MT**: MTR based on short echo time imaging; T1sat: T1 of saturated pool; **UTE-MTR**: MTR based on ultrashort echo time imaging; **QSM**: quantitative susceptibility mapping; **Others: rSPF**: relative semi-solid proton fraction from an 3D ultrashort echo time (UTE) sequence within an appropriate water suppression condition; **T1w/T2w**: ratio of image intensity in a T1-weighted vs T2-weighted acquisition.

Reference	Modality	Metrics	T	Resolution (mm)	Tissue state	Temperature
Turati et al. (2015)	MT	MP('f')	7	0.1 x 0.1 x 0.6 mm	in vivo	body temperature
Underhill et al. (2011)	MT	MP('BPF'), MTR	3	0.3 0.3 0.3 mm	in vivo	body temperature
van der Voorn et al. (2011)	MT	MTR	1.5	not reported (3 mm sections)	fixed	room temperature (20-22°C)
West et al. (2018)	MT	MP (BPF')	15.2	.150 x .150 x .150 mm	perfusion fixed	not reported
Righart et al. (2017)	Other	T1w/T2w	1.5	1 x 1 x 1.5 mm	in situ	not reported
Soustelle et al. (2019)	Other	rSPF	7	0.152 x 0.152 x 0.750 mm	perfusion fixed	room temperature (20°C)
Argyridis et al. (2014)	QSM	Susceptibility	9.4	0.06 mm isotropic	perfusion fixed	not reported
Hametner et al. (2018)	QSM	Susceptibility	7	0.43 x 0.43 x 0.65 mm	in situ	room temperature (20 deg)
Lodygensky et al. (2012)	QSM	Susceptibility	9.4	slice thickness of 0.5 mm, in-plane resolution between 0.0625 mm isotropic and 0.105 mm isotropic	in vivo	body temperature
Pol et al. (2019)	QSM	Susceptibility	9.4	not reported	in vivo	body temperature
Soni et al. (2020)	QSM	Susceptibility	9.4	0.1 x 0.1 x 0.3 mm	in vivo	body temperature (36.537°C)
Stüber et al. (2014)	QSM	Susceptibility	7	0.070 mm isotropic	fixed	not reported
Bagnato et al. (2018)	Relaxometry	R2*	7	0.7 mm isotropic	fixed	room temperature
Bot et al. (2004)	Relaxometry	T1, T2	4.7	0.007 x 0.007 x 1 mm	fixed	not reported
Chen et al. (2017)	Relaxometry	MWF	7	0.07 x 0.07 x 05 mm	perfusion fixed	not reported
Hakkarainen et al. (2016)	Relaxometry	RAFFn, T1, T2	9.4	0.156 x 0.156 x .35 mm	perfusion fixed	not reported
Hametner et al. (2018)	Relaxometry	T1, R2*	7	0.43 x 0.43 x 0.65 mm	in situ	room temperature (20 deg)
Jelescu et al. (2016)	Relaxometry	T2	7	0.112 x 0.112 x 0.8 mm	in vivo	body temperature
Kelm et al. (2016)	Relaxometry	MWF	15.2	0.15 x 0.15 x 0.15 mm	perfusion fixed	17 ± 0.5 °C
Kozlowski et al. (2008)	Relaxometry	MWF	7	0.078 x 0.078 x 1 mm	perfusion fixed	not reported
Kozlowski et al. (2014)	Relaxometry	MWF	7	0.117 x 0.117 x 1 mm (ex vivo) 1 x 1 x 1.5 mm (in vivo)	in vivo (relaxometry) and fixed (DWI)	body temperature / not reported
Laule et al. (2008)	Relaxometry	MWF	7	0.234 x 0.234 x 1 mm	fixed	not reported
Laule et al. (2006)	Relaxometry	MWF	1.5	in plane resolution not reported, 3mm thick slices	fixed	20°C
Laule et al. (2011)	Relaxometry	MWF	1.5 and 7	0.586 x 0.586 x 3 mm (1.5T), 0.234 x 0.234 x 1mm (7T)	fixed	not reported
Lehto et al. (2017)	Relaxometry	RAFF4	7	0.125 x 0.125 x 0.5 mm	in vivo	body temperature
Mottershead et al. (2003)	Relaxometry	PD, T1, T2	7	Single 1.5 mm slices, in plane resolution between 0.055 and 0.068 mm	fresh	29-38°C

Continued on next page

Table 2: **Information on MRI imaging of the assessed validation studies.** Provided are imaging modality, field strength in Tesla (T), imaging resolution in terms of voxel size, and the tissue state and temperature during scanning. **Acronyms: Imaging: DWI: Diffusion-weighted imaging**: **AK**: axial kurtosis; **AI**: Anisotropy Index (tADC/lADC); **DBSI-RD**: diffusion basis spectrum imaging - based radial diffusivity; **DK**: diffusion kurtosis metrics; **DT**: diffusion tensor metrics; **FA**: fractional anisotropy (from diffusion tensor model); **lADC**: longitudinal apparent diffusion coefficient (not modelled with tensor); **MD**: mean diffusivity (from diffusion tensor model); **MK**: mean kurtosis; **RD**: radial / transverse diffusivity (from diffusion tensor model); **RK**: radial kurtosis; **SDI**: diffusion standard deviation index; **tADC**: transverse apparent diffusion coefficient (not modelled with tensor); **Relaxometry**: **MWF**: myelin water fraction; **R1**: longitudinal relaxation rate; **R2***: effective transverse relaxation rate; **RAFF4**: Relaxation Along a Fictitious Field in the rotating frame of rank 4; **T1**: longitudinal relaxation time; **T2**: transverse relaxation time; **T2***: effective transverse relaxation time; **MT: magnetisation transfer**: **BPF**: bound pool fraction; **F**: pool size ratio; **Fb**: macromolecular proton fraction; **ih-MTR**: MTR from inhomogeneous MT; **M0b**: fraction of magnetization that resides in the semi-solid pool and undergoes MT exchange; **MP**: macromolecular pool; **MPF**: macromolecular proton fraction; **MTR**: magnetisation transfer ratio; **PSR**: Macromolecular-to-free-water pool-size-ratio; **STE-MT**: MTR based on short echo time imaging; T1sat: T1 of saturated pool; **UTE-MTR**: MTR based on ultrashort echo time imaging; **QSM**: quantitative susceptibility mapping; **Others: rSPF**: relative semi-solid proton fraction from an 3D ultrashort echo time (UTE) sequence within an appropriate water suppression condition; **T1w/T2w**: ratio of image intensity in a T1-weighted vs T2-weighted acquisition.

Reference	Modality	Metrics	T	Resolution (mm)	Tissue state	Temperature
Oakden et al. (2015)	Relaxometry	MWF	1.5	0.2 0.2 x 1 mm	in vivo / fixed	body temperature
Odrobina et al. (2005)	Relaxometry	MWF, T1, T2	1.5	not reported	fixed	room temperature (20°C)
Pun et al. (2005)	Relaxometry	T1, MWF	1.5	not reported	fresh	20 °C
Reeves et al. (2015)	Relaxometry	T1, T2, T2*	9.4	0.136 x 0.136 x 0.5 mm	fixed	not reported
Schmierer et al. (2010)	Relaxometry	T1	9.4	0.117 x 0.156 x 1 mm	fixed	not reported
Schmierer et al. (2004)	Relaxometry	T1	1.5	0.938 x 0.938 x 5 mm	fresh	mean 25.0°C (SD: 2.9°C)
Schmierer et al. (2007a)	Relaxometry	T1	1.5	0.938 x 0.938 x 5 mm	fresh	room temperature
Schmierer et al. (2008)	Relaxometry	T1, T2	1.5	0.94 x 0.94 x 5 mm	fresh / fixed	22.5°C
Seewann et al. (2009)	Relaxometry	T1	1.5	1 x 1 x 3 mm	fixed	not reported
Soustelle et al. (2019)	Relaxometry	MWF	7	0.1 x 0.1 x 0.750 mm	perfusion fixed	room temperature (20°C)
Stüber et al. (2014)	Relaxometry	R1, R2*	7	R1: 0.1 or 0.2 mm isotropic, R2*: 0.2 mm isotropic	fixed	not reported
Thiessen et al. (2013)	Relaxometry	T1	7	0.098 0.098 0.750 mm	perfusion fixed	room temperature
Warntjes et al. (2017)	Relaxometry	MWF, R1	3	0.7 x 0.7 x 4 mm	in situ	mean: 7.8° (SD: 3.1°C)
Webb et al. (2003)	Relaxometry	MWF	1.5	unclear	fresh	20°C
West et al. (2018)	Relaxometry	MWF	15.2	.150 x .150 x .150 mm	perfusion fixed	not reported

Table 3: **Information on histology of the assessed validation studies.** Provided are information in tissue preparation, histological slice thickness, the histological method for myelin (and whether iron and axons were also considered) and the obtained histological metric. **Acronyms: AMG**: Autometallographic myelin stain; **LFB**: Luxol fast blue stain; **MA**: fraction of myelinated axons; **MAB238**: Anti-oligodendrocyte immunohistochemistry; **MBP**: Anti-myelin-basic-protein immunohistochemistry; **MSF**: myelin sheath fraction; **MST**: myelin sheath thickness; **MVF**: myelin volume fraction; **PAS**: periodic acid-Schiff; **PIXE**: proton-induced X-ray emission; **PLP**: Anti-proteolipid-protein immunohistochemistry.

Reference	Tissue preparation	Slice thickness	Myelin histology	Iron	Axons	Histology Metric
Abe et al. (2019)	cryosectioning	25 μm	PLP	-	+	staining fraction
Aojula et al. (2016)	perfusion fixation, cryosectioning	15 μm	MBP	-	-	staining fraction
Argyridis et al. (2014)	not reported	2 μm	LFB	+	-	contrasted and normalised luminance
Bagnato et al. (2018)	not reported	10 μm	LFB and PLP	+	-	staining intensity
Beckmann et al. (2018)	paraffin embedding	3 μm	LFB	-	-	staining intensity
Bot et al. (2004)	not reported	5 μm	LFB	-	+	normalised staining intensity
Chandran et al. (2012)	paraffin embedding	5 μm	LFB, MBP	-	-	staining intensity
Chang et al. (2017b)	CLARITY	3D	MBP	-	-	staining intensity
Chang et al. (2017a)	CLARITY	3D	MBP	-	-	normalised staining intensity
Chen et al. (2017)	ultrasectioning	1 μm	EM	-	+	staining fraction
Choi et al. (2015)	cryosectioning	50 μm	Gold chloride	-	+	staining intensity
Duhamel et al. (2019)	perfusion fixation, cryosectioning	20 μm	PLP-GFP fluorescence	-	-	normalised (background) staining intensity

Continued on next page

Table 3: **Information on histology of the assessed validation studies.** Provided are information in tissue preparation, histological slice thickness, the histological method for myelin (and whether iron and axons were also considered) and the obtained histological metric. **Acronyms: AMG**: Autometallographic myelin stain; **LFB**: Luxol fast blue stain; **MA**: fraction of myelinated axons; **MAB238**: Anti-oligodendrocyte immunohistochemistry; **MBP**: Anti-myelin-basic-protein immunohistochemistry; **MSF**: myelin sheath fraction; **MST**: myelin sheath thickness; **MVF**: myelin volume fraction; **PAS**: periodic acid-Schiff; **PIXE**: proton-induced X-ray emission; **PLP**: Anti-proteolipid-protein immunohistochemistry.

Reference	Tissue preparation	Slice thickness	Myelin histology	Iron	Axons	Histology Metric
Fatemi et al. (2011)	perfusion fixation, paraffin embedding / cryosectioning	20 μm / 40 μm	LFB, MBP	-	+	staining intensity
Fjær et al. (2013)	fixation, paraffin embedding	7 μm	PLP	-	-	staining fraction
Fjær et al. (2015)	fixation, paraffin embedding	7 μm	LFB, PLP	+	-	manual scoring, staining fraction
Gareau et al. (2000)	not reported	5 μm	Solochrome-R-cyanine	-	-	score of estimated 'myelin pallor'
Grussu et al. (2017)	paraffin embedding	10 μm	PLP	-	+	staining fraction
Hakkarainen et al. (2016)	cryosectioning	30 μm	Gold chloride	+	-	normalised staining intensity
Hametner et al. (2018)	fixation, cutting, paraffin embedding	10 μm	LFB	+	-	staining intensity
Janve et al. (2013)	paraffin embedding	10 μm	LFB	-	-	normalised staining intensity
Jelescu et al. (2016)	perfusion fixation, nanosectioning	2.36 nm	EM	-	+	staining fraction
Jespersen et al. (2010)	cryosectioning	40 μm	AMG	-	+	staining intensity
Jito et al. (2008)	perfusion fixation, semithin sectioning	750 nm	Toluidine blue	-	+	staining fraction
Kelm et al. (2016)	ultrasectioning	70 nm	EM	-	+	staining fraction
Khodanovich et al. (2017)	cryosectioning	10 μm	LFB	-	-	staining intensity
Khodanovich et al. (2019)	cryosectioning	10 μm	MBP	-	-	staining fraction
Kozlowski et al. (2008)	cryosectioning	20 μm	LFB, MBP	-	+	staining intensity (MBP), inverse staining intensity (LFB)
Kozlowski et al. (2014)	cryosectioning	20 μm	MBP, Eriochromecyanine	-	+	normalised staining intensity
Laule et al. (2008)	paraffin embedding	10 μm	LFB	-	-	staining intensity
Laule et al. (2006)	paraffin embedding	10 μm	LFB	-	-	staining intensity
Laule et al. (2011)	paraffin embedding	10 μm	LFB, MBP	-	+	normalised staining intensity
Lehto et al. (2017)	cryosectioning	30 μm	Gold chloride	-	-	staining intensity
Lodygensky et al. (2012)	perfusion fixation, cryosectioning	50 μm	Black Gold II	+	-	staining intensity
Martirosyan et al. (2016)	paraffin embedding	15 μm	LFB	-	-	staining intensity, percentage difference between injury epicenter to control animals
Moll et al. (2011)	cryosectioning	30 μm	MBP	-	+	staining intensity
Mollink et al. (2019)	paraffin embedding	6 μm	PLP	-	+	staining fraction
Mottershead et al. (2003)	fixation, paraffin embedding	10 μm	LFB	-	+	staining fraction
Oakden et al. (2015)	paraffin embedding	10 μm	LFB	-	-	visual classification of severity of pathology
Odrobina et al. (2005)	epon-araldite embedding	1 μm	Toluidine blue	-	-	staining fraction
Peters et al. (2019)	fixation and sectioning	13 μm	LFB	-	-	staining intensity
Pol et al. (2019)	perfusion fixation and cryosectioning	16 μm	Solochrome	-	-	staining intensity
Praet et al. (2018)	fixation, paraffin embedding	5 μm	MBP	-	-	staining intensity
Pun et al. (2005)	fixation, ultrasectioning	1 μm	Toluidine blue	-	+	staining fraction
Reeves et al. (2015)	not reported	7 μm	MBP	-	+	staining intensity
Righart et al. (2017)	paraffin embedding	10 μm	PLP	-	+	staining intensity
Schmierer et al. (2010)	paraffin embedding	5 μm	MBP	-	+	inverse staining intensity
Schmierer et al. (2004)	paraffin embedding	not reported	LFB	-	+	inverse staining intensity
Schmierer et al. (2007a)	fixation, paraffin embedding	not reported	LFB	-	+	inverse staining intensity
Schmierer et al. (2007b)	fixation, paraffin embedding	9.4±3.6 μm	LFB	-	+	inverse staining intensity
Schmierer et al. (2008)	paraffin embedding	5000 μm	LFB	-	+	inverse staining intensity

Continued on next page

Table 3: **Information on histology of the assessed validation studies.** Provided are information in tissue preparation, histological slice thickness, the histological method for myelin (and whether iron and axons were also considered) and the obtained histological metric. **Acronyms: AMG**: Autometallographic myelin stain; **LFB**: Luxol fast blue stain; **MA**: fraction of myelinated axons; **MAB238**: Anti-oligodendrocyte immunohistochemistry; **MBP**: Anti-myelin-basic-protein immunohistochemistry; **MSF**: myelin sheath fraction; **MST**: myelin sheath thickness; **MVF**: myelin volume fraction; **PAS**: periodic acid-Schiff; **PIXE**: proton-induced X-ray emission; **PLP**: Anti-proteolipid-protein immunohistochemistry.

Reference	Tissue preparation	Slice thickness	Myelin histology	Iron	Axons	Histology Metric
Schwartz et al. (2005)	epon immersion	1 μm	Toluidine blue	-	+	staining fraction (MVF, MST)
Seehaus et al. (2015)	cryosectioning	60 μm	Gallyas	-	-	staining intensity
Seewann et al. (2009)	paraffin embedding	10 μm	LFB, PAS, PLP	-	+	inverse staining intensity
Soni et al. (2020)	perfusion fixation, paraffin embedding	10 μm	MBP	-	-	manual scoring of immunoreactivity
Soustelle et al. (2019)	sectioning	60 μm	MBP	-	-	normalized staining intensity
Stüber et al. (2014)	cryosectioning	30 μm	PIXE	+	-	model-based estimation of myelin volume fraction
Sundberg et al. (2010)	perfusion fixation, cryosectioning	35 μm	MAB328	-	+	staining fraction
Takagi et al. (2009)	fixation and sectioning	80 nm	EM	-	+	staining fraction
Thiessen et al. (2013)	epon embedding	1 μm	EM	-	+	staining fraction (MA, MSF)
Tu et al. (2016)	cryosections	10 μm	MBP	-	+	normalised staining intensity
Turati et al. (2015)	perfusion fixation, cryosectioning	10 μm	Black Gold II, MBP	-	-	staining intensity
Underhill et al. (2011)	perfusion fixation, paraffin embedding	5 μm	LFB	-	+	normalized staining intensity
van der Voorn et al. (2011)	paraffin embedding	7 μm	LFB	-	+	inverse staining intensity
van Tilborg et al. (2018)	paraffin embedding	8 μm	MBP	-	-	staining fraction
Wang et al. (2009)	perfusion fixation, cryosectioning	10 μm	LFB	-	+	staining intensity
Wang et al. (2015)	paraffin embedding	5 μm	LFB	-	+	staining fraction
Warntjes et al. (2017)	fixation	4 μm	LFB	-	-	staining intensity
Webb et al. (2003)	fixation, crosssectioning	1 μm	Toluidine blue	-	+	staining fraction
Wei et al. (2013)	cryosectioning	50000 μm	Gold chloride	-	-	inverse staining intensity
West et al. (2018)	ultrasectioning	0.07 μm	EM	-	+	staining fraction
Yano et al. (2018)	cryosectioning	25 μm	PLP	-	-	staining fraction

Table 4: **Information on the employed statistical approach of the assessed validation studies.** Provided are information on the nature of data points that entered the correlation analysis, the method for coregistration between MRI and histology, sample size (number of subjects per group, total number of subjects, and number of data points for the analysis), the type of variance that was modelled in the statistical analysis, the reported statistic, and whether the study was selected for our meta-analysis (MA). **Acronyms: BS**: between-subject; **WS**: within-subject

Reference	Correlation	Coregistration	N (Subjects/group)	N (Total subjects)	N (ROIs)	Statistical Design	Statistics	MA
Abe et al. (2019)	ROI to ROI	No coregistration	8	8	96 (in total across subjects)	Mixed (not modelled)	Pearson	- (statistical design)
Aojula et al. (2016)	ROI to ROI	No coregistration	4 kaolin-only, 6 kaolin + PBS (control intervention), 5 kaolin + decorin, 4 controls,	19	2	Between-subject	Spearman	BS Pearson

Continued on next page

Table 4: **Information on the employed statistical approach of the assessed validation studies.** Provided are information on the nature of data points that entered the correlation analysis, the method for coregistration between MRI and histology, sample size (number of subjects per group, total number of subjects, and number of data points for the analysis), the type of variance that was modelled in the statistical analysis, the reported statistic, and whether the study was selected for our meta-analysis (MA). **Acronyms: BS**: between-subject; **WS**: within-subject

Reference	Correlation	Coregistration	N (Subjects/group)	N (Total subjects)	N (ROIs)	Statistical Design	Statistics	MA
Argyridis et al. (2014)	ROI to ROI	Not reported	18	18	3 (pooled into 1 weighted measure)	Between-subject	Not reported (likely linear regression)	BS Pearson
Bagnato et al. (2018)	ROI to ROI	No coregistration	7	7	429 (in total across subjects)	Within-subject (mixed, modelled)	Pearson; linear regression	WS Pearson
Beckmann et al. (2018)	ROI to ROI	Not reported	5	20	1	Between-subject	Pearson	BS Pearson
Bot et al. (2004)	ROI to ROI	No coregistration	11 MS, 2 controls	13	222 (in total across subjects)	Mixed (unclear)	Spearman; linear regression	- (statistical design)
Chandran et al. (2012)	ROI to ROI	Not reported	5 to 6 per group	20	1	Between-subject	Linear regression	BS Pearson
Chang et al. (2017b)	ROI to ROI	Linear registration (12 dof affine)	4	4	14 (in total across subjects)	Mixed (not modelled)	Spearman; linear regression	- (statistical design)
Chang et al. (2017a)	ROI to ROI	Unclear	4	4	14 (in total across subjects)	Mixed (not modelled)	Spearman	- (statistical design)
Chen et al. (2017)	ROI to ROI	NA	3 to 6 per group	14	1	Between-subject	not reported	- (statistical measure)
Choi et al. (2015)	ROI to ROI	Linear registration (affine, in Matlab)	2 MPS brains, 1 control	3	16	Within-subject	Correlation coefficient type not reported; Multivariate linear regression	- (statistical measure)
Duhamel et al. (2019)	ROI to ROI	No coregistration	3	3	8 (7 ROIs + 1 ”ROI” of relative GM/WM contrast)	Mixed (not modelled)	Pearson; linear regression	- (statistical design)
Fatemi et al. (2011)	ROI to ROI	Not reported	29 ischemic injury, 33 controls	61	25 (in total across subjects)	Unclear (likely mixed, not modelled)	Linear regression	- (statistical design)
Fjær et al. (2013)	ROI to ROI	No coregistration	6 controls, 48 cuprizone at varying stages	54	4	Between-subject	Linear regression	BS Pearson
Fjær et al. (2015)	ROI to ROI	Not reported	6	24	5	Between-subject	Linear regression	BS Pearson
Gareau et al. (2000)	unclear (likely ROI to ROI)	No coregistration	6x4 EAE at different time points, 6 controls	24	1	Between-subject	Spearman	BS Spearman
Grussu et al. (2017)	ROI to ROI	Landmark-based non-linear registration	2 MS, 2 controls	4	48 (in total across subjects)	Mixed (not modelled)	Pearson; linear regression	- (statistical design)
Hakkarainen et al. (2016)	ROI to ROI	Not reported	5 to 6 per group	5	60 (in total across subjects)	Not reported (likely mixed, not modelled)	Pearson	- (statistical design)

Continued on next page

Table 4: **Information on the employed statistical approach of the assessed validation studies.** Provided are information on the nature of data points that entered the correlation analysis, the method for coregistration between MRI and histology, sample size (number of subjects per group, total number of subjects, and number of data points for the analysis), the type of variance that was modelled in the statistical analysis, the reported statistic, and whether the study was selected for our meta-analysis (MA). **Acronyms: BS**: between-subject; **WS**: within-subject

Reference	Correlation	Coregistration	N (Subjects/group)	N (Total subjects)	N (ROIs)	Statistical Design	Statistics	MA
Hametner et al. (2018)	ROI to ROI	No coregistration	6	6	2870 (in total across subjects) for R2*, 2809 (in total across subjects) for T1	Within-subject (mixed, modelled)	Linear regression	WS Pearson
Janve et al. (2013)	ROI to ROI	Manual coregistration	8 LPS, 1 control	9	6	Mixed (not modelled)	Pearson	- (statistical design)
Jelescu et al. (2016)	ROI to ROI	NA	2 x 12 Cuprizone, 10 controls	34	1	Between-subject	Partial Spearman (weight as covariate)	BS Spearman
Jespersen et al. (2010)	ROI to ROI	No coregistration	3	3	8	Mixed (not modelled)	Pearson	- (statistical design)
Jito et al. (2008)	ROI to ROI	NA	6	36	1	Between-subject	Linear regression	BS Pearson
Kelm et al. (2016)	ROI to ROI	No coregistration	4 TSC, 3 Rictor, 5 Controls	12	6	Mixed (not modelled)	not reported	- (statistical design)
Khodanovich et al. (2017)	ROI to ROI	No coregistration	7 cuprizone, 7 controls	14	6	Within-subject (mixed, modelled)	Pearson; linear regression	WS Pearson
Khodanovich et al. (2019)	ROI to ROI	No coregistration	4 demyelination, 5 remyelination, 4 controls	13	1	Between-subject	Pearson; linear regression	BS Pearson
Kozlowski et al. (2008)	ROI to ROI	No coregistration	6 at 3 weeks post injury, 4 at 8 weeks post injury, 6 controls (controls not used in correlation)	6 and 4 depending on the analysis	3	Mixed (not modelled)	Pearson	- (statistical design)
Kozlowski et al. (2014)	ROI to ROI	No coregistration	8	16	1	Between-subject	Pearson	- (no result)
Laule et al. (2008)	ROI to ROI	Landmark-based linear registration	3 MS patients, 10 samples	3	22-30 per sample	Within-subject	Not reported (likely linear regression)	- (N = 3)
Laule et al. (2006)	ROI to ROI	Landmark-based linear registration	13	13	23-45 per slice	Within-subject	Not reported (likely linear regression)	WS Pearson
Laule et al. (2011)	ROI to ROI	Landmark-based linear registration	9 MS patients, 23 samples	9	Not reported	Within-subject	Not reported (likely linear regression)	WS Pearson

Continued on next page

Table 4: **Information on the employed statistical approach of the assessed validation studies.** Provided are information on the nature of data points that entered the correlation analysis, the method for coregistration between MRI and histology, sample size (number of subjects per group, total number of subjects, and number of data points for the analysis), the type of variance that was modelled in the statistical analysis, the reported statistic, and whether the study was selected for our meta-analysis (MA). **Acronyms: BS**: between-subject; **WS**: within-subject

Reference	Correlation	Coregistration	N (Subjects/group)	N (Total subjects)	N (ROIs)	Statistical Design	Statistics	MA
Lehto et al. (2017)	ROI to ROI	No coregistration	21	21	12 and 2 depending on the analysis	Mixed (not modelled)	Pearson	- (statistical design)
Lodygensky et al. (2012)	ROI to ROI	Not reported	13 (across different ages)	13	2	Between-subject	Linear regression; Spearman mentioned but not reported	BS Pearson
Martirosyan et al. (2016)	ROI to ROI	No coregistration	15 from different types of SCI, 3 controls	18	1	Between-subject	Pearson	BS Pearson
Moll et al. (2011)	ROI to ROI	No coregistration	4	4	12 (in total across subjects)	Mixed (not modelled)	Spearman	- (statistical design)
Mollink et al. (2019)	ROI to ROI	No coregistration	14 ALS and 5 Controls	19	1	Between-subject	Pearson	BS Pearson
Mottershead et al. (2003)	ROI to ROI	No coregistration	4 MS patients, 1 control	5	108 (in total across subjects)	Mixed (not modelled)	Spearman	- (statistical design)
Oakden et al. (2015)	ROI to ROI	Manual coregistration	8 for acute group, 8 for chronic group	16	Variable and only specified for significant correlations	Mixed (not modelled)	Pearson	- (statistical design)
Odrobina et al. (2005)	unclear (likely ROI to ROI)	No coregistration	Not reported	Not reported	Not reported (likely 2)	Unclear (likely mixed, not modelled)	Not reported	- (statistical design)
Peters et al. (2019)	voxel-wise	3D registration	3	3 (1 reported)	not reported	Within-subject	Spearman	- (N = 1)
Pol et al. (2019)	ROI to ROI	No coregistration	13 Teliflunomide, 12 controls	25	3	Between-subject	Pearson	BS Pearson
Praet et al. (2018)	ROI to ROI and voxelwise	3D stacking of histology, non-linear registration	48 Alzheimer's model, 32 wild type	80	1	Between-subject	Pearson (Bonferroni corrected for multiple comparisons); Bayesian multivariate linear regression	BS Pearson
Pun et al. (2005)	unclear	Unclear	62 Tellerium, 34 controls	96	1	Between-subject	not reported	- (statistical measure)
Reeves et al. (2015)	ROI to ROI	Manual coregistration	12 epilepsy, 1 control	13	3-4 (43 in total across subjects)	Mixed (not modelled)	Spearman; linear regression	- (statistical design)

Continued on next page

Table 4: **Information on the employed statistical approach of the assessed validation studies.** Provided are information on the nature of data points that entered the correlation analysis, the method for coregistration between MRI and histology, sample size (number of subjects per group, total number of subjects, and number of data points for the analysis), the type of variance that was modelled in the statistical analysis, the reported statistic, and whether the study was selected for our meta-analysis (MA). **Acronyms: BS**: between-subject; **WS**: within-subject

Reference	Correlation	Coregistration	N (Subjects/group)	N (Total subjects)	N (ROIs)	Statistical Design	Statistics	MA
Righart et al. (2017)	ROI to ROI	No coregistration	9	9	5 per patient; 36 in total across subjects	Mixed (not modelled)	Generalized estimating equations	- (statistical design)
Schmierer et al. (2010)	ROI to ROI	Manual coregistration	21	2	2	Mixed (not modelled)	Linear regression	- (statistical design)
Schmierer et al. (2004)	ROI to ROI	Manual coregistration	19	20	68 (in total across subjects)	Within-subject (mixed, modelled)	Pearson	WS Pearson
Schmierer et al. (2007a)	ROI to ROI	Manual coregistration	35	35	129 (in total across subjects)	Within-subject (mixed, modelled)	Pearson	WS Pearson
Schmierer et al. (2007b)	ROI to ROI	No coregistration	16	16	44-51 depending on the analysis	Within-subject (mixed, modelled)	Pearson	WS Pearson
Schmierer et al. (2008)	ROI to ROI	No coregistration	15	15	40-44 (in total across subjects)	Mixed (unclear whether modelled)	Linear regression	- (statistical design)
Schwartz et al. (2005)	ROI to ROI	No coregistration	3	3	6	Mixed (unclear whether modelled)	Pearson; linear regression	- (statistical design)
Seehaus et al. (2015)	voxel-wise	3D affine	1	1	voxel-wise (62782)	Within-subject	Pearson	- (N = 1)
Seewann et al. (2009)	ROI to ROI	Manual coregistration	10	10	42 (in total across subjects)	Mixed (modelled, but unclear whether result refers to within or between subject variance)	Pearson; Spearman mentioned but not reported	- (statistical design)
Soni et al. (2020)	ROI to ROI	No coregistration	36 TBI, 6 controls; correlation only for TBI group	36	3	Between-subject	Spearman	BS Spearman
Soustelle et al. (2019)	ROI to ROI	No coregistration	7 cuprizone-fed, 8 controls	15	3 (1/animal in each correlation)	Between-subject	Spearman	BS Spearman
Stüber et al. (2014)	pixel-wise	Landmark-based registration	3	3	pixel-wise	Within-subject	Pearson; linear regression (univariate and multiple)	- (N = 3)
Sundberg et al. (2010)	ROI to ROI	No coregistration	15 injury, 9 control (two groups analysed separately)	15, 9 depending on the analysis	3	Mixed (not modelled)	Pearson	- (statistical design)
Takagi et al. (2009)	sample to sample	NA	10	70	1	Unclear	Pearson	- (statistical design)

Continued on next page

Table 4: **Information on the employed statistical approach of the assessed validation studies.** Provided are information on the nature of data points that entered the correlation analysis, the method for coregistration between MRI and histology, sample size (number of subjects per group, total number of subjects, and number of data points for the analysis), the type of variance that was modelled in the statistical analysis, the reported statistic, and whether the study was selected for our meta-analysis (MA). **Acronyms: BS**: between-subject; **WS**: within-subject

Reference	Correlation	Coregistration	N (Subjects/group)	N (Total subjects)	N (ROIs)	Statistical Design	Statistics	MA
Thiessen et al. (2013)	ROI to ROI	No coregistration	5 cuprizone, 5 controls	10	1	Between-subject (both within groups and pooled across groups) and within-group results have n = 5 rather than n = 10	Spearman	BS Spearman
Tu et al. (2016)	ROI to ROI	No coregistration	5	25	7	Unclear (likely mixed, not modelled)	Pearson	- (statistical design)
Turati et al. (2015)	ROI to ROI	No coregistration	15 per mouse strain	15 for each analysis	1	Between-subject (pooled across groups)	Spearman	BS Spearman
Underhill et al. (2011)	ROI to ROI	No coregistration	5 and 4	9	1	Within-subject (mixed, modelled)	Pearson	WS Pearson
van der Voorn et al. (2011)	ROI to ROI	Manual coregistration	15 patients, 5 controls	20	55 (in total across subjects)	Mixed (not modelled)	Pearson, linear regression analysis	- (statistical design)
van Tilborg et al. (2018)	unclear	Unclear	6	12	3	Between-subject	Linear regression	BS Pearson
Wang et al. (2009)	ROI to ROI	No coregistration	15 at different ages	15	Unclear (likely 2)	Unclear (likely mixed, not modelled)	Pearson	- (statistical design)
Wang et al. (2015)	voxel-wise	Landmark-based linear coregistration (rigid body, based on 13 landmarks)	3	3	80 voxels (subset of total voxels selected for analysis)	Within-subject	Spearman	- (N = 3)
Warntjes et al. (2017)	pixel-wise	Manual coregistration by rotation, translation, and scaling	12	12	voxel-wise	Within-subject (mixed, modelled)	Spearman; linear regression	WS Spearman
Webb et al. (2003)	ROI to ROI	Manual coregistration	6	18	2	Mixed (not modelled)	Linear regression	- (statistical design)
Wei et al. (2013)	ROI to ROI	3D stacking of histological images and linear coregistration	1	1	20	Within-subject	Pearson; linear regression	- (N = 1)
West et al. (2018)	unclear	NA	3 per group, 6 controls	15	unclear (likely 4)	Mixed (not modelled)	Linear regression	- (statistical design)
Yano et al. (2018)	ROI to ROI	No coregistration	18 cuprizone treated, 3 controls	21	1	Between-subject	Pearson	BS Pearson

Table 5: Information on the statistical results of the assessed validation studies.

Reference	Modality	Result	Linear equation
Abe et al. (2019)	DWI	FA: r = .32 (p = .012), RD: r = -.41 (p = .001), AD: r = -.11 (p = .39)	NA
Aojula et al. (2016)	DWI	AD: r = .159, p = .541 (CC); r = -.360, p = 0.155 (PVWM); FA: r = .091, p = .729 (CC); r = .346, p = .174 (PVWM); MD: r = -.031, p = .903 (CC); r = -.495, p = .043 (PVWM); RD: r = -.115, p = .66 (CC); r = -.458, p = .064 (PVWM)	NA

Continued on next page

Table 5: Information on the statistical results of the assessed validation studies.

Reference	Modality	Result	Linear equation
Argyridis et al. (2014)	DWI	MD: R2 = .003, p = .46	MD = -0.09 * myelin + 0.69
Chandran et al. (2012)	DWI	FA: R2 = .27, p = .016 (LFB), R2 = .50, p = .0003 (MBP); RD: R2 = .34, p = .005 (MBP), R2 = 0.05; p = .31 (LFB)	not reported
Chang et al. (2017b)	DWI	AD: p = .61; FA: p < .01; RD: p = .83	Not reported
Chang et al. (2017a)	DWI	AD: r = .0385; FA: r = .446, p <.05; MD: r = -.114; RD: r = -.195	NA
Choi et al. (2015)	DWI	FA: p = 1.9 10^{-5} (control), p = .989 and p = .776 (MPS); RD: p < .0001 (control), p = .917 and p = .459 (MPS)	Not reported
Grussu et al. (2017)	DWI	AD: r = .75, p < .001; FA: r = .42, p = .025; MD: r = .67; p < .001; RD: r = .61; p < .001	See paper
Janve et al. (2013)	DWI	AD: r = .03 (lesion), r = -.08 (combined); FA: r = .16 (lesion), r = .27 (combined); RD: r = -.40 (lesion), r = -.49 (combined)	NA
Jelescu et al. (2016)	DWI	RD: r = -.71, p = .0004; RK: r = .14, p = .5638	NA
Jespersen et al. (2010)	DWI	FA: r = .78, p = 7 10^{-8}	NA
Jito et al. (2008)	DWI	FA: r = .856, p < .0001	not reported
Kelm et al. (2016)	DWI	FA: not significant; MD: R2 = .35; RD: R2 = .37; MK: R2 = .48; AK: not significant; RK: r = .49	NA
Kozlowski et al. (2008)	DWI	AD: r = -.27, p = .07) for LFB at 3 weeks, r = .24 (p = .16) for LFB at 8 weeks, r = -.35 (p = .02) for MBP at 3 weeks, r = .03 (p = .88) for MBP at 8 weeks; FA: r = .33 (p = .02) for LFB at 3 weeks, r = .72 (p < .001) for LFB at 8 weeks, r = -.66 (p < .0001) for MBP at 3 weeks, r = .22 (p = .19) for MBP at 8 weeks; MD: r = -.60 (p < .001) for LFB at 3 weeks, r = -.57 (p < .001) for LFB at 8 weeks, r = .26 (p = .08) for MBP at 3 weeks, r = -.19 (p = .26) for MBP at 8 weeks; RD: r = -.49 (p < .001) for LFB at 3 weeks, r = -.71 (p < .001) for LFB at 8 weeks, r = .51 (p < .001) for MBP at 3 weeks, r = -.21 (p = .22) for MBP at 8 weeks	NA
Kozlowski et al. (2014)	DWI	not reported	NA
Lehto et al. (2017)	DWI	AD: r = .714, p < .001 (CC), r = .279, p = .223 (DTT); FA: r = .662, p < .001 (C), r = .438, p = .053 (DTT); MD: r = .708, p < .001 (CC), r = .112, p = .610 (DTT); RD: r = .257, p = .048 (CC), r = -.079, p = .739 (DTT)	NA
Martirosyan et al. (2016)	DWI	FA: r = -.94 (p = .0001)	NA
Moll et al. (2011)	DWI	AD: r = -.41 (p = .004); FA: r = .38 (p = .008); MD: r = -.58 (p < .0001), RD: r = -.52 (p = .0002)	NA
Mollink et al. (2019)	DWI	FA: r = .52, p = .03	NA
Mottershead et al. (2003)	DWI	ADC: r = -.45 (p = .001); SDI: r = .51 (p < .001)	NA
Oakden et al. (2015)	DWI	AD: r = .39; RD: r = .57	NA
Peters et al. (2019)	DWI	FA: r = .10 (CT), r = .09 (Tuber), r = .45 (Perituber), r = .19 (WM), r = .70 (All); MD: r = .04 (CT), r = .07 (Tuber), r = -.63 (Perituber), r = -.60 (WM), r = -.85 (All)	NA
Pol et al. (2019)	DWI	FA: r = -.240, p = .675 (caudal medial, controls), r = -.130, p = .802 (rostral lateral, controls), r = -.147, p = .785 (rostral medial, controls), r = -.122, p = .825 (caudal medial, Teliflunomide), r = -.265, p = .643 (rostral lateral, Teliflunomide), r = .017, p = .981 (rostral medial, Teliflunomide); MD: r = .592, p = .202 (caudal medial, controls), r = .377, p = . 438 (rostral lateral, controls), r = .221, p = .675 (rostral medial, controls), r = -.083, p = .896 (caudal medial, Teliflunomide), r = -.237, p = . 667 (rostral lateral, Teliflunomide), r = .069, p = .903 (rostral medial, Teliflunomide);	NA
Praet et al. (2018)	DWI	AD: r = -.479, p = .0362; AK: r = .376 (p = .4851); FA: r = -.560 (p = .0025); MD: r = -.175, p = 1; MK: r = .504 (p = .0173); RD: r = .272, p = 1; RK: r = .518 (p = .0110); Voxelwise statistics: DT: r = .5545, DK: r = .4509, DT/DK: .6374	Not reported
Schmierer et al. (2007b)	DWI	FA: r = -.79, p < .001; MD: r = .68, p < .001	NA
Schmierer et al. (2008)	DWI	AD: r = .53 (unfixed) and r = .8 (fixed) ; FA: r = -.78 (unfixed) and r = -.83 (fixed); MD: r = .73 (unfixed) and r = .78 (fixed); RD: r = .74 (unfixed) and .81 (fixed), all p < .01	Not reported

Continued on next page

Table 5: Information on the statistical results of the assessed validation studies.

Reference	Modality	Result	Linear equation
Schwartz et al. (2005)	DWI	AI: r = -.58, p = .0125 (MVF) , r = 0.19, p = 0.4536 (MST); lADC: r = -.48, p = .0437 (MVF), r = .43, p = .0717 (MST); tADC: r = -.74, p = .0004 (MVF), r = .34, p = .1701 (MST)	Not reported
Seehaus et al. (2015)	DWI	FA: r = .487, MD: r = -.542, RD: r = -.554	NA
Seewann et al. (2009)	DWI	ADC and LFB: r = .28; ADC and PLP: r = .16 ; FA and LFB: r = -.63, p < .01; FA and PLP: r = -.35, p < .05	NA
Soustelle et al. (2019)	DWI	RD: All: r = -.7, p < .05 (medial CC), r = -.63, p > .05 (lateral CC); r = -.21, p > .05 (CT); Controls only: r = .38 (medial CC), r = .40 (lateral CC), r = .45 (CT); Cuprizone only: r = -.07 (medial CC), r = .14 (lateral CC), r = .79 (CT);	NA
Sundberg et al. (2010)	DWI	AD: r = .4825, p = .0216 (lateral), r = .4799, p = .0282 (dorsal); FA: r = -.8235, p = .0382 (dorsal column of caudal zone);	NA
Takagi et al. (2009)	DWI	FA: r = .2387, p = .4796 (myelin sheath density); r = -.6941, p = .0178 (Myelin sheath thickness)	NA
Thiessen et al. (2013)	DWI	AD: r = -.75, p < .001 (MSF); r = -.89, p < .01 (MA); FA: r = .84, p < .01 (MSF), r = .7, p < .001 (MA); MD: r = .81, p < .01 (MSF); r = .81, p < .01 (MA); RD: r = .87, p < .01 (MSF); r = -.77, p < .001 (MA); for correlation separately for each group see paper	NA
Tu et al. (2016)	DWI	AD: r = -.01; FA: r = .13, MD: r = -.17, RD: r = -.46 (p < .01); MTR-3.5: r = .38 (p < .01), MTR-20: r = .28	NA
van der Voorn et al. (2011)	DWI	ADC: r = - .637 (p < .01); FA: r = .766 (p < .01)	Myelin = -13.7 + 143.9 * FA
van Tilborg et al. (2018)	DWI	FA: R2 = .341, p = .046 (M1 motor), R2 = .334, p = .049 (M2 motor), R2 = .428, p = .021 (sensory)	not reported
Wang et al. (2009)	DWI	FA: r = .681, p < .01; RD: r = 0.528, p < .01; AD and Trace showed no significant correlation	NA
Wang et al. (2015)	DWI	DBSI-RD: r = - .84 (p < .0001), r = -.42 (p = .039), r = -.82 (p < .0001)	Available for each subject - see paper
Wei et al. (2013)	DWI	FA: r = .12, p = .68 (WM), r = .61, p = .32 (GM). RD: r = .55, p = .15 (WM), r = .594, p = .28 (GM)	Not reported
Yano et al. (2018)	DWI	FA: r = .87, p = 3.89 x $10^{-}7$; MD: r = -.73, p = 1.54 x $10^{-}4$; RD: r = -.91, p = 1.04 x $10^{-}8$	NA
Beckmann et al. (2018)	MT	MTR: R2 = .7669 (p < .0001)	NA
Bot et al. (2004)	MT	MTR: r = -.76, p < .001	See paper
Duhamel et al. (2019)	MT	ih-MTR: between r = .93 and r = .98, p < .0001, depending on the specific sequence	See paper
Fatemi et al. (2011)	MT	MTR: R2 = .695, p < .0001; correlations with LFB not reported	not reported
Fjær et al. (2013)	MT	MTR: R2 = .338, p < .0001 (medial CC); R2 = .426, p < .0001 (lateral CC); R2 = .208, p < .001 (dGM); R2 = .052, p = .1048 (CT)	intercept = -3.002, slope 0.004 (medial CC); intercept -2.910, slope = 0.002 (lateral CC); intercept = -1.414, slope = 0.018 (dGM); intercept = -1.273, slope = 0.347 (CT)
Fjær et al. (2015)	MT	MTR and LFB: not reported. MTR and PLP: R2 = 0.01, p = .63 (CC), R2 = .08, p = .2 (dGM), R2 < .01, p = .97 (CT);	not reported
Gareau et al. (2000)	MT	MTR: R2 = .45, p < .001	NA
Hakkarainen et al. (2016)	MT	MTR: r = .34 (p < .001)	NA
Janve et al. (2013)	MT	MP: r = .87 (lesion), r = .85 (all)	NA
Jelescu et al. (2016)	MT	MTR: r = .32, p = .1821	NA
Kelm et al. (2016)	MT	not reported	NA
Khodanovich et al. (2017)	MT	MP: R2 = .897, p < .001 (all); r = .870, p = .007 (control); r = .927, p = 0.002 (Cuprizone)	MP = 0.124 * myelin + 6.278
Khodanovich et al. (2019)	MT	MP: r = .90, p < .001 (CC); r = .83, p < .001 (Caudate/putamen); r = .80, p < .001 (Hippocampus); r = .88, p < .001 (CT)	MP = 0.4-0.6 * myelin + 6.2-7.84 (slope and intercept vary depending on anatomical area)

Continued on next page

Table 5: Information on the statistical results of the assessed validation studies.

Reference	Modality	Result	Linear equation
Lehto et al. (2017)	MT	MTR: r = .741, p < .001 (CC), r = .719, p = .001 (DTT); T1sat: r = -.741, p < .001 (CC), r = -.705, p = .001 (DTT)	NA
Moll et al. (2011)	MT	MTR: r = 0.63 (p < .0001)	NA
Mottershead et al. (2003)	MT	MTR: r = -.29 (p = .075)	NA
Odrobina et al. (2005)	MT	MTR: r = .56 ± .22 (p < .0005); MP: r = .77 ± .15 (p < .0001)	NA
Reeves et al. (2015)	MT	MTR: p < .005	Not reported
Schmierer et al. (2010)	MT	MTR: r = .52, p = .02	Not reported
Schmierer et al. (2004)	MT	MTR: r = -.84, p < .001	NA
Schmierer et al. (2007a)	MT	MP: r = -.80, p < .001; MTR: r = -.84, p < .001	NA
Schmierer et al. (2008)	MT	MP: r = -.72 (unfixed) and r = -.86 (fixed), all p < .01; MTR: r = -.83 (unfixed) and r = -.68 (fixed)	Not reported
Seewann et al. (2009)	MT	MTR and LFB: r = -.63, p < .01; MTR and PLP: r = -.27	NA
Soustelle et al. (2019)	MT	MP: All: r = .87, p < .05 (medial CC), r = .91, p < .05 (lateral CC); r = .89, p < .05 (CT); Controls only: MP: r = .36 (medial CC); r = .55 (lateral CC); r = .86 (CT); Cuprizone only: r = .64 (medial CC); r = .82 (lateral CC); r = .25 (CT);	NA
Thiessen et al. (2013)	MT	MP: r = .93; p < .001 (MSF); r = .81, p < .01 (MA); MTR: r = .72, p < .001 (MSF); r = .73, p < .001 (MA); for correlation separately for each group see paper; Pearson: r = 0.98	Myelin = 5.37 * MP - 0.25
Turati et al. (2015)	MT	MP: in C57BL/6 mice: r = .743, p = .002 (BGII); r = .589, p = .023 (MBP); in SJL/J mice: r = .586, p = .024 (BGII) and r = .536, p = .042 (MBP)	NA
Underhill et al. (2011)	MT	MP: r = .99 (p < .001), r = .91, p = .03 (just GM), r = .95, p = .0047 (just WM); MTR: r = .96 (p < .001), r = .93, p = .021 (just GM), r = -.21, p = .79 (just WM); for results on four-parameter estimation of MP, see paper	MP = 0.21 * myelin + 3.9
van der Voorn et al. (2011)	MT	MTR: r = .727 (p < .01)	Not reported
West et al. (2018)	MT	MWF: R2 = .70	MWF = .056 * myelin 0.014
Righart et al. (2017)	Other	NA	Not reported
Soustelle et al. (2019)	Other	rSPF: r = .86, p < .05 (medial CC), r = .76, p < .05 (lateral CC), r = .52, p > .05 (CT)	NA
Argyridis et al. (2014)	QSM	susceptibility: R2 = .93, p = .004	Susceptibility = -0.14 * myelin + 0.14
Hametner et al. (2018)	QSM	Susceptibility: r = -.352; for results separate for tissue class see paper	Susceptibility = 0.008758 0.000196 * myelin
Lodygensky et al. (2012)	QSM	Susceptibility: R2 = .673, p < .0003 (AC); R2 = .76, p < .0001 (CC)	Susceptibility = - 0.0107 * myelin - 0.0017 (AC); Susceptbility = - 0.015 * myelin + 0.004 (CC)
Pol et al. (2019)	QSM	Susceptibility: r = -.190, p = .723 (caudal medial, controls), r = -.214, p = .675 (rostral lateral, controls), r = .005, p = .987 (rostral medial, controls), r = .601, p = .141 (caudal medial, Teliflunomide), r = .243, p = .658 (rostral lateral, Teliflunomide), r = .211, p = .695 (rostral medial, Teliflunomide)	NA
Soni et al. (2020)	QSM	susceptibility: R2 = .1, p = .02 (only reported for 1 out of 3 ROIs)	NA
Stüber et al. (2014)	QSM	Susceptability: p < .001	Susceptibility: slope: - 0.069 ± 0.002, intercept: - 0.025 ± 0.001
Bagnato et al. (2018)	Relaxometry	R2* and LFB: r = -.09, p < .01 (NAWM); r = -.05, p < .01 (DWMI); r = .99, p<.01 (WM-Ls); r = .83, p < .01 (thalamus); r = .82, p < .01 (dGM); shadow plaques: not significant; R2 = 0.014 (all ROIs combined); R2* and PLP: only CT reported, r = 0.57 p < .01	R2* = 0.02 * myelin
Bot et al. (2004)	Relaxometry	T1: r = .71 (p < .001); T2: r = .77 (p < .001)	See paper
Chen et al. (2017)	Relaxometry	MWF: r = .823, p < .001	NA

Continued on next page

Table 5: Information on the statistical results of the assessed validation studies.

Reference	Modality	Result	Linear equation
Hakkarainen et al. (2016)	Relaxometry	T1: r = .77; T2: r = .18; TRAFF 1: r = .33; T RAFF2: r = .47; T RAFF3: r = .56; T RAFF4: r = .83; T RAFF5: r = .84 (all p < .001)	NA
Hametner et al. (2018)	Relaxometry	R2*: r = .027; T1 (log): r = -.583; for results separate for tissue class see paper	R2* = 44.7355 0.002942 * myelin; T1 (log) = 7.2738 0.001928 * myelin
Jelescu et al. (2016)	Relaxometry	T2: r = -.64, p = .0024	NA
Kelm et al. (2016)	Relaxometry	not reported	NA
Kozlowski et al. (2008)	Relaxometry	MWF: r = .64 (p < .001) for LFB at 3 weeks, r = .88 (p < .001) for LFB at 8 weeks, r = -.28 (p = .07) for MBP at 3 weeks, r = .15 (p = .40) for MBP at 8 weeks	NA
Kozlowski et al. (2014)	Relaxometry	MWF: r = .63, p = .0047 (EC, in vivo), r = .74, p = .0001 (EC, ex vivo), r = .64, p = .05 (MBP, in vivo), r = .63, p = .038 (MBP, ex vivo)	NA
Laule et al. (2008)	Relaxometry	MWF: R2 between .56 and .95 (mean: .78), all p < .0001; R2 between 0 and .79 (mean R2 = .43, p < .0001) just in WM ROIs	NA
Laule et al. (2006)	Relaxometry	MWF: R2 between .45 and .92 (mean .67), all p < .0001; mean R2 = .29 (p < .0001) just in WM ROIs	NA
Laule et al. (2011)	Relaxometry	MWF and LFB: R2 between .48 and .95 (mean: .74); MWF and MBP: R2 between .15 and .47 (mean: .31)	NA
Lehto et al. (2017)	Relaxometry	RAFF4: r = -.742, p < .0001 (CC), r = -.745, p = .001 (DTT)	NA
Mottershead et al. (2003)	Relaxometry	PD: r = -.72 (p < .001); T1: r = -.78 (p < .001); T2: r = -.75 (p < .001)	NA
Oakden et al. (2015)	Relaxometry	MWF: r = -.06	NA
Odrobina et al. (2005)	Relaxometry	MWF: r = .71 ± .16 (p < .001); T1: r = -.88 ± 12 (p < .0001); T2: r = -.91 ± .08 (p < .0001)	NA
Pun et al. (2005)	Relaxometry	MWF: r = .77, T1: r = .88	NA
Reeves et al. (2015)	Relaxometry	T1: R2 = .270 (p < .005); T2: R2 = .235 (p < .005); T2*: p < .05	Not reported
Schmierer et al. (2010)	Relaxometry	T1: not reported	Not reported
Schmierer et al. (2004)	Relaxometry	T1: r = .70, p < .001	NA
Schmierer et al. (2007a)	Relaxometry	T1: r = .69, p < .001	NA
Schmierer et al. (2008)	Relaxometry	T1: r = .77 (unfixed) and r = .89 (fixed), T2: r = .82 (unfixed) and r = .92 (fixed), all p < .01	Not reported
Seewann et al. (2009)	Relaxometry	T1 and LFB: r = .68, p < .01; T1 and PLP: r = .41, p < .05	NA
Soustelle et al. (2019)	Relaxometry	MWF: r = .68, p < .05 (medial CC), r = .69, p < .05 (lateral CC), r = .20, p > .05 (CT)	NA
Stüber et al. (2014)	Relaxometry	R1/R2*: p < .001; R2*: r = 0.805	R1: slope: 1.066 ± 0.016, intercept: 1.132 ± 0.009; R2*: slope: 38.59 ± 0.50, intercept: 45.86 ± 0.29,
Thiessen et al. (2013)	Relaxometry	T1: r = -.66, p < .001 (MSF); r = -.73 (MA); for correlation separately for each group see paper	NA
Warntjes et al. (2017)	Relaxometry	R1: r = .63 ± .12; MWF: r = 0.74 ± 0.11	R1: slope: 0.065 ± 0.009; intercept: 1.19 ± 0.10
Webb et al. (2003)	Relaxometry	MWF: r = .59, p < .001 (all fibres); r = .75, p < .0001 (healthy fibres)	MWF: slope = 0.4 ± 0.1 (all fibres), slope = 0.7 ± 0.1 (healthy fibres)
West et al. (2018)	Relaxometry	MWF: R2 = .66	MWF = .056 * myelin 0.014
